# Eye-tracking technology in identifying visualizers and verbalizers: data on eye-movement differences and detection accuracy

**DOI:** 10.1016/j.dib.2019.104447

**Published:** 2019-08-29

**Authors:** Zhanni Luo, Yu Wang

**Affiliations:** a Educational Studies and Leadership, University of Canterbury, New Zealand; b Human Interface Technology Lab, University of Canterbury, New Zealand

**Keywords:** Visualizers, Verbalizers, Learning styles, Detection, Eye-tracking technology

## Abstract

Data in this article revealed the eye movement differences of visualizers and verbalizers in viewing four pictures-in-text by analyzing gaze path and fixation data (fixation duration, fixation counts and the average time on each fixation). After imported the documents into Tobii eye-tracker, authors triggered participants’ natural reading habits, recorded their eye movement data, and predicted participants as visualizers or verbalizers based on the Felder and Silverman Learning Style Model (FSLSM). Comparing the predictions with self-report results tested by the Index of Learning Styles (ILS) questionnaire, authors got the accuracy results of using eye-tracking technology in identifying visualizers and verbalizers. The data revealed natural preferences of people with different styles, and it can be used in future studies in the field of adaptive learning systems, individual differences, neuroscience in reading habits, and individualized instruction.

**Table:** Specifications Table

Subject	*Education; Human-Computer Interaction; Developmental and Educational Psychology*
Specific subject area	*Educational technologies and learning style detection*
Type of data	*Table and image*
How data was acquired	*Survey and Tobii eye-tracker*
Data format	*Raw and analyzed data*
Parameters for data collection	*Gaze paths, fixation counts, fixation duration and average time on each fixation*
Description of data collection	*First, set visual and verbal areas with the tool provided by Tobii eye-tracker; secondly, prepare four pictures-in-texts that contain different arrangements of images and texts; thirdly, ask participants to read the four pictures-in-text; fourthly, output the gaze plot paths and fixation data recorded by Tobii eye-tracker; fifthly, ask participants to fill the Index of Learning Style (ILS) questionnaire and get participants' styles (either visual or verbal) according to the traditional pencil-and-paper approach.*
Data source location	*City: Kuala Lumpur* *Country: Malaysia* *GPS: 2°56′42.00″ N 101°52′26.40″ E*
Data accessibility	*On Mendeley Data* https://doi.org/10.17632/xvt962wptp*.3*

**Table:** 

**Value of the data** • Data in this study revealed natural differences of people with different styles (visual or verbal). It can be used in the study of adaptive learning systems, which aims to provide different learners with individualized materials that suit them best; • As to learning style theories, it is criticized that there is no credible evidence that learning styles exist [1], and the related studies have not been grounded in credible psychological concepts [2]. Data in this study provides a bridge between learning style hypothesis and scientific theories. • Data in this study showed that pictures with different image-and-text combinations can affect the accuracy of learning-style identification. It supports further explorations on the factors affecting the accuracy of learning-style identification; • Data and research design in this study can be referenced in future studies using eye-tracking technologies to identify other learning styles, such as intuitive/sensory learners and active/reflective learners.

## 1 Data

**Fig. 1:**
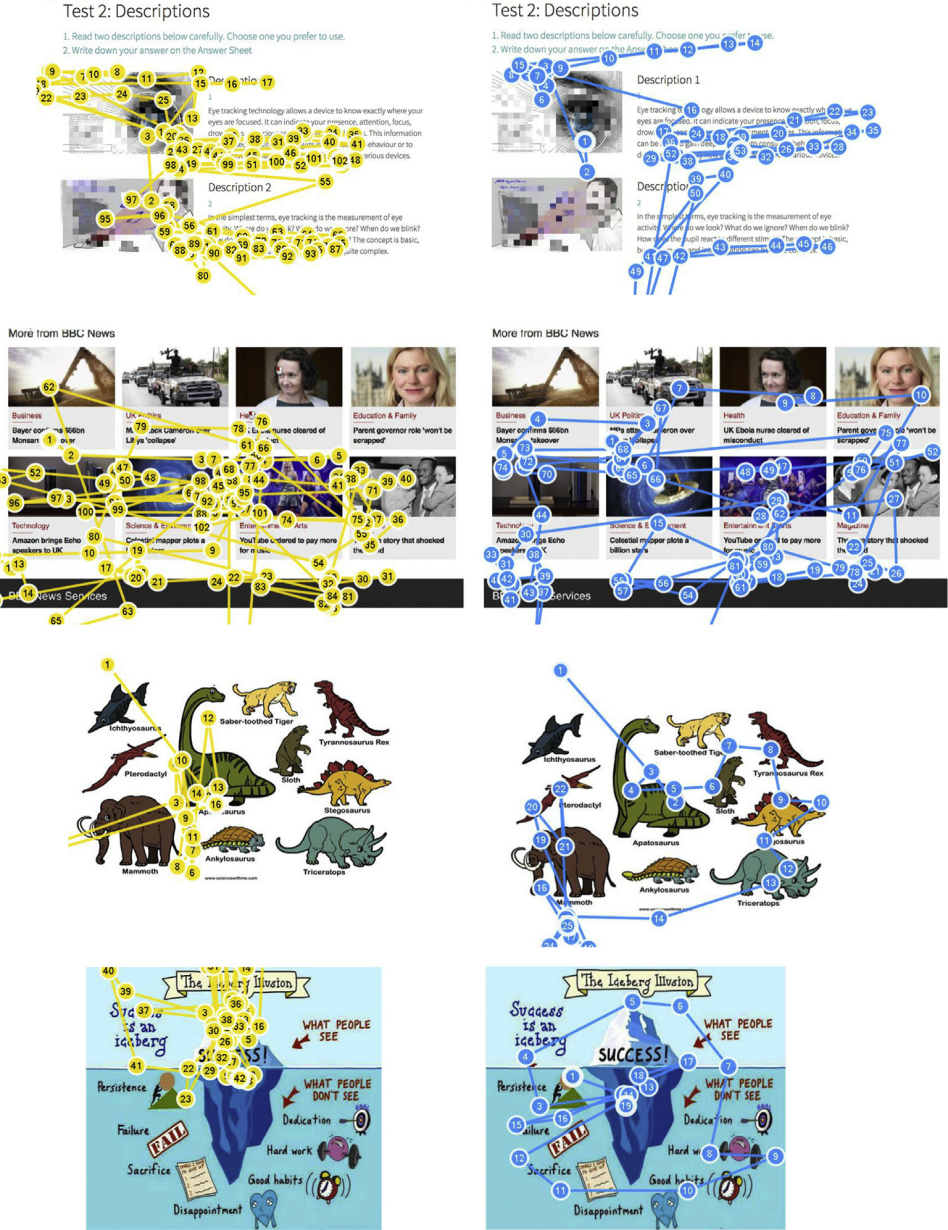
Gaze plot paths of a visualizer (left) and a verbalizer (right).

**Fig. 2:**
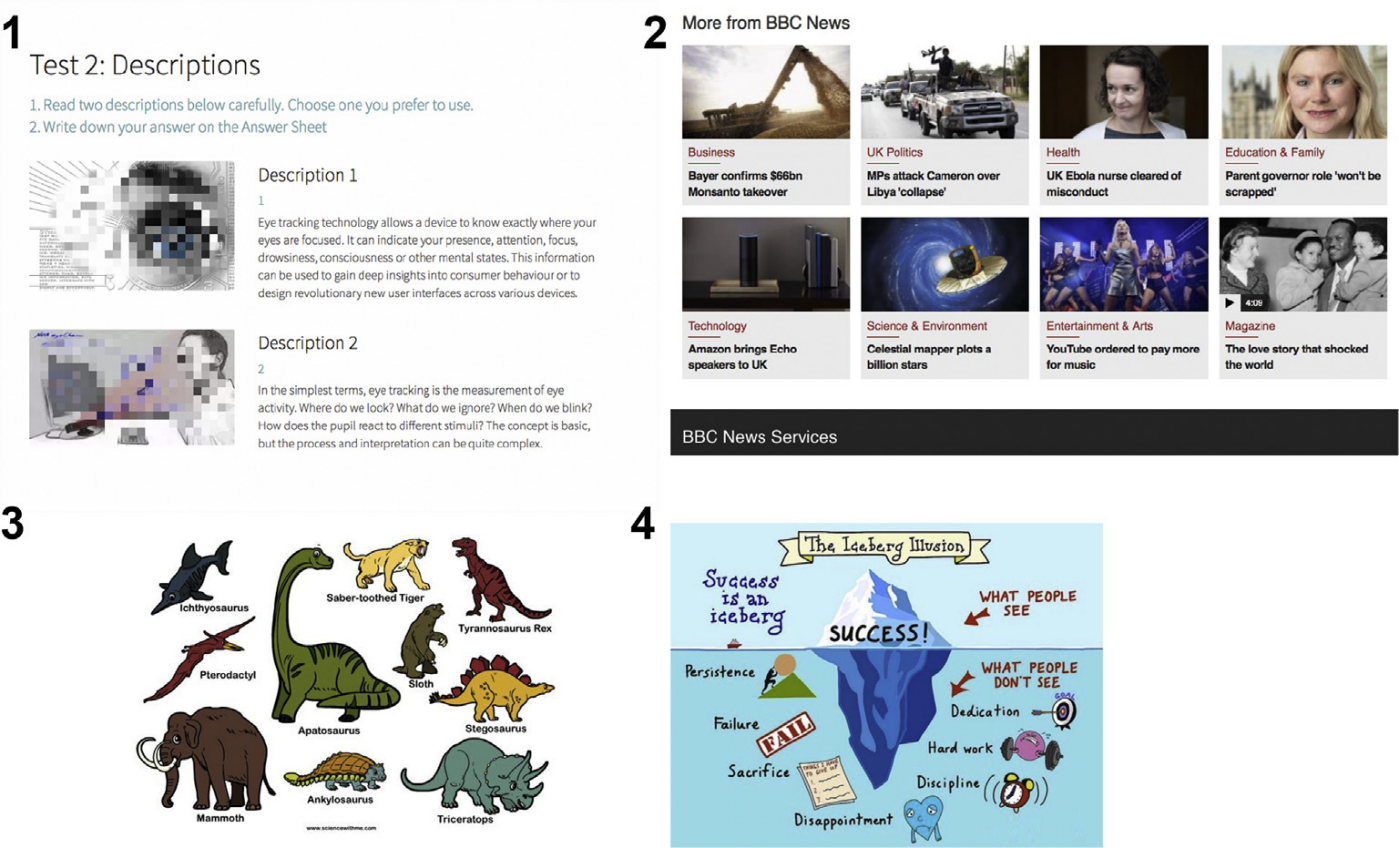
Reading materials for the current quasi-experiment.

The shared data are recordings from a quasi-experiment in which the eye movement data of different participants, either visualizers or verbalizers, were recorded. In viewing the given materials (see Fig. 2), participants’ eye movement data were recorded, including gaze paths (see Fig. 1) and fixation data (see Table 1, Table 2, Table 3). By comparing the prediction based on eye movement data and the results of self-reported styles tested by ILS questionnaire, the accuracy of identifying visualizers and verbalizers by eye-tracking technology was calculated (see Table 4). The original data is available on Mendeley Data [3].

**Table 1:** Fixation data of visualizers and verbalizers on visual and verbal AOIs in the four pictures-in-text.

Category	Documents	Visual AOIs			Verbal AOIs		
		Fixation count (n)	Mean (s)	Fixation Duration (s)	Fixation count (n)	Mean (s)	Fixation Duration (s)
Visualizers	Doc 1	12.17	0.24	2.90	73.83	0.28	21.17
	Doc 2	38.58	0.28	11.00	55.92	0.33	18.85
	Doc 3	7.50	0.87	5.69	4.67	0.45	2.14
	Doc 4	6.83	1.02	6.13	5.83	0.59	3.85
	Average	16.27	0.60	6.43	35.06	0.41	11.50
Verbalizers	Doc 1	38.58	0.28	11.00	55.92	0.33	18.85
	Doc 2	71.70	0.50	21.79	92.21	0.51	28.57
	Doc 3	13.05	1.25	8.80	7.19	0.67	3.13
	Doc 4	11.44	1.56	9.54	10.58	1.18	7.35
	Average	33.69	0.89	12.78	41.47	0.67	14.48

**Table 2:** Fixation data of visualizers and verbalizers on visual and verbal AOIs in the four pictures-in-text .

AOI	Measures	Visualizers	Verbalizers	Difference (visual-verbal)
Visual AOIs	Fixation count (n)	16.27	33.69	−6.35
	Fixation Duration (s)	6.43	12.78	−0.29
	Mean (s)	0.60	0.89	−6.41
Verbal AOIs	Fixation count (n)	35.06	41.47	−2.98
	Fixation Duration (s)	11.50	14.48	−0.26
	Mean (s)	0.41	0.67	−6.35

**Table 3:** Fixation data on visual AOIs and Verbal AOIs based on the document with high prediction accuracy (Doc 4).

AOI	Measures	Visualizers	Verbalizers	Difference (visual-verbal)
Visual AOIs	Fixation count (n)	6.83	11.44	−4.61
	Fixation Duration (s)	6.13	9.54	−3.41
	Mean (s)	1.02	1.56	−0.54
Verbal AOIs	Fixation count (n)	5.83	10.58	−4.75
	Fixation Duration (s)	3.85	7.35	−3.5
	Mean (s)	0.59	1.18	−0.59

**Table 4:** Accuracy of visualizer/verbalizer identification of the four pictures-in-text.

Document	Visual AOIs	Verbal AOIs	Accuracy in general
Document 1	86%	25%	55%
Document 2	57%	50%	54%
Document 3	93%	13%	53%
Document 4	79%	75%	77%

Learners are categorized as visualizers or verbalizers according to their favoured methods of receiving external information, either by images or texts. Namely, visualizers prefer visual information while verbalizers prefer textual information [4]. This dataset aims to present the potential of using eye-tracking technology in identifying visualizers and verbalizers, as well as its accuracy.

Fig. 1 presents the different patterns of eye-movement paths of a visualizer (left) and a verbalizer (right) in four sets of pictures-in-text. The four illustrations on the left side were generated by participant No. 22, and the four on the right side were generated by participant No. 10.

Table 1, Table 2 show the fixation data by visualizers and verbalizers in visual and verbal AOIs (Area of Interest) for the four documents used in this research, with the former one enlisting the data for each document and the latter one presenting the average data of the four documents.

The units of measurement for fixation counts, fixation duration and mean are numbers (n), seconds (s) and seconds (s) respectively. ‘Difference (visual-verbal)’ refers to ‘the data generated by visualizers’ minus ‘the data generated by verbalizers’.

To guarantee the accuracy, researchers also used the same instrument to test Document 4, which reported a comparatively high level of accuracy (see Table 4). The data reported the same tendency as Table 1 did (see Table 3).

Table 4 is the accuracy of visualizer/verbalizer identification of the four pictures-in-text, including the accuracy on visual AOIs, verbal AOIs and the accuracy in general.

## 2 Experimental design, materials, and methods

### 2.1 Materials

Four pictures-in-text were chosen for the current study, with each one containing different arrangements of images and text: the spatial distribution of image and text in Document 1 is right-to-left, and that in Document 2 is up-to-down. Pictures and text in Document 3 and 4 are presented in pairs, but the two documents are different in size and color. Meanwhile, the images in Document 1 and 2 are text-unrelated, while that in document 3 and 4 are text-related (see Fig. 2).

All of these documents were selected from existing online materials. Document 1 was edited by researchers with definitions from Wikipedia and two decorative images from Google Picture. In this study, the exact images in Document 1 were blurred due to copyright concerns. Document 2 was a screenshot from BBC News in 2016 without copyright conflicts. The other two documents (Document 3 and 4) were reprinted with permission from the copyright owner Sciencewithme! [5] and Duckworth [6] respectively.

### 2.2 Research design and methods

The two main research methods adopted in the current experiment are quasi-experimentation with the use of eye-tracking technology and survey study with the ILS questionnaire (Index of Learning Style) by Felder and Soloman [7].

Firstly, authors imported research materials into Tobii eye-tracker and marked the visual AOIs and verbal AOIs. In the Tobii eye-tracking system, researchers can draw lines to cover the areas they want to focus on, which is called Area of Interest (AOI), and the system will record data within the marked AOIs.

Secondly, authors asked participants to do easy tasks such as “choose one description you like from the two enlisted below” (Task 1), “choose two pieces of news you are interested in” (Task 2), and “view each picture and choose one item you like. You can write or draw” (Task 3 and 4). The purpose of providing easy tasks is to trigger natural reading habits, which is essential for quasi-experiments.

Then, the Tobii eye-tracker recorded participants' gaze paths and fixation data. Gaze paths, recorded as gaze plot patterns or eye movement patterns, represent the visual route taken by the user's eyes as they move across the screen. Gaze paths illustrate users' coverage of visual and verbal areas and help reveal the trends of user learning styles. Fixation data includes the fixation counts, fixation duration, and the average time for each fixation (means).

After the quasi-experiment, participants were asked to fill the Index of Learning Styles questionnaire (ILS). ILS contains eleven questions for visual/verbal learner detection, with two options in each question. Option A refers to a visual preference and option B indicates a verbal one. In the data calculation, option A is marked with −1 point, while option B is marked with +1 point. The total amount of eleven answers were added, and a negative number refers to a visualizer while a positive number refers to a verbalizer.

**Fig. 3:**
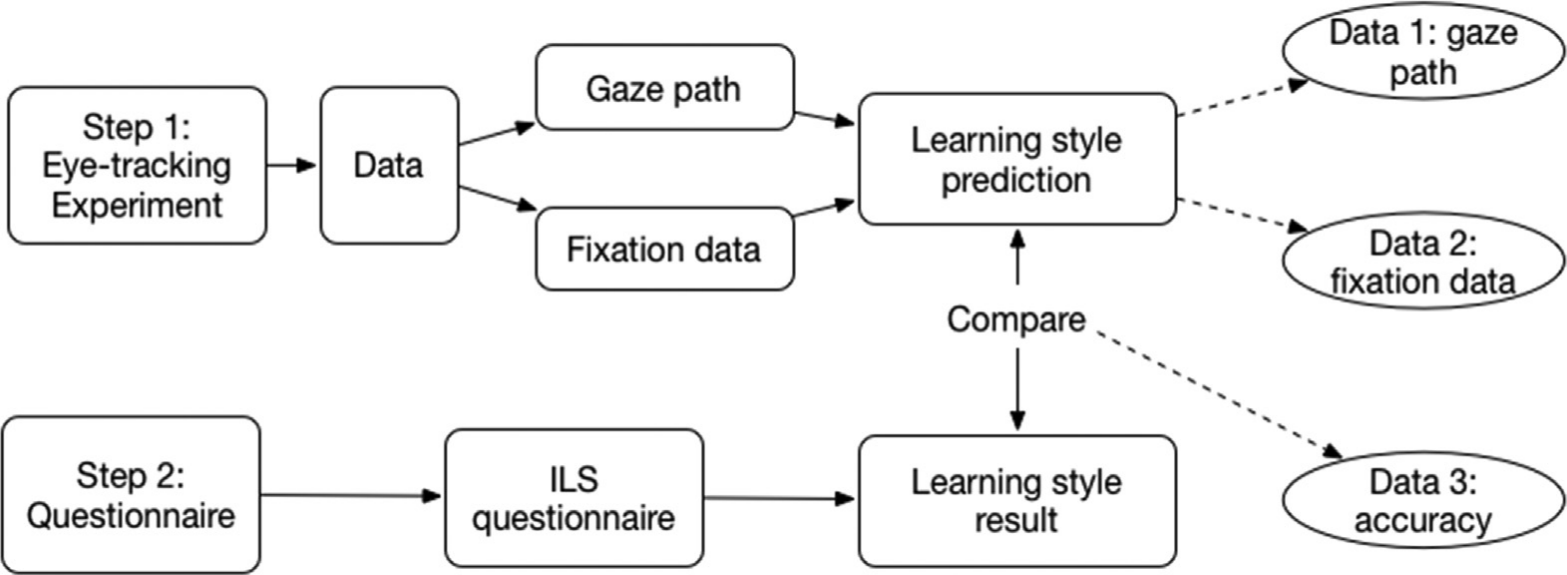
Research design.

By analyzing the gaze paths and fixation data, authors categorized participants as visualizers or verbalizers. Compared to the prediction with the self-report learning style results, authors of this study got the accuracy rate of using eye-tracking technology in identifying visualizers or verbalizers. The research process is shown in Fig. 3.

In the whole process, three types of data were generated: the gaze path, the fixation data, and the prediction accuracy. Gaze paths are presented as individual illustrations for each participant; Fixation data is generated in a table, including three key measures: fixation duration, fixation counts and the average time for each fixation (means); Prediction accuracy should be calculated by the authors, by comparing the predication based on eye movement data with the self-report results tested by ILS questionnaires.
