# Corrigendum to “Eye-tracking technology in identifying visualizers and verbalizers: Data on eye-movement differences and detection accuracy” [Data in Brief 26 (2019) 104447]

**DOI:** 10.1016/j.dib.2019.105003

**Published:** 2019-12-13

**Authors:** Zhanni Luo, Yu Wang

**Affiliations:** a Educational Studies and Leadership, University of Canterbury, New Zealand; b Human Interface Technology Lab, University of Canterbury, New Zealand

The authors regret that the wrong version of Table 1, Table 2, Table 3 were used originally, resulting in incorrect data being published. The corrected tables have been mentioned below.

**Table 1:** Fixation data of visualizers and verbalizers on visual and verbal AOIs in the 4 sets of pictures-in-text (data for each document).

Category	Documents	Visual AOI			Verbal AOI		
		Fixation count (n)	Mean (s)	Duration (s)	Fixation count (n)	Mean (s)	Duration (s)
Visualizer	Doc 1	14.07	0.25	3.58	76.14	0.29	22.44
	Doc 2	38.21	0.52	11.10	53.57	0.64	17.97
	Doc 3	19.21	0.25	5.02	11.83	0.32	3.97
	Doc 4	18.21	0.32	5.33	6.79	0.26	1.82
	Average	22.43	0.33	6.26	37.08	0.38	11.55
Verbalizer	Doc 1	8.75	0.21	1.90	54.13	0.31	16.54
	Doc 2	44.13	0.58	13.45	56.38	0.63	17.38
	Doc 3	17.50	0.33	5.55	12.88	0.31	4.22
	Doc 4	17.38	0.29	5.03	5.50	0.33	1.90
	Average	21.94	0.35	6.48	32.22	0.40	10.01

**Table 2:** Fixation data of visualizers and verbalizers on visual and verbal AOIs in the four sets of pictures-in-text.

AOI	Measure	Visualizer	Verbalizer	Difference (visual-verbal)
Visual AOIs	Fixation count (n)	89.71	87.75	+1.96
	Mean (s)	1.33	1.42	−0.08
	Fixation duration (s)	25.03	25.94	−0.90
Verbal AOIs	Fixation count (n)	148.33	128.88	+19.46
	Mean (s)	1.52	1.58	−0.07
	Fixation duration (s)	46.20	40.04	+6.17

**Table 3:** Fixation data on visual AOIs and Verbal AOIs based on the document with high prediction accuracy (Doc 4).

AOI	Measure	Visualizer	Verbalizer	Difference (visual-verbal)
Visual AOIs	Fixation count (n)	18.21	17.38	+0.84
	Mean (s)	0.32	0.29	+0.03
	Fixation duration (s)	5.33	5.03	+0.30
Verbal AOIs	Fixation count (n)	6.79	5.50	+1.29
	Mean (s)	0.26	0.33	−0.06
	Fixation duration (s)	1.82	1.90	−0.08

The authors would like to apologise for any inconvenience caused.

