# Supplementary material for: Eye-tracking technology in identifying visualizers and verbalizers: data on eye-movement differences and detection accuracy
Source: Data Brief. 2019 Aug 29;26:104447. doi: 10.1016/j.dib.2019.104447 (PMC6811880; doi:10.1016/j.dib.2019.104447)
Supplement: Multimedia component 1 [file mmc1.zip › Data Data in Brief/1 Experiment Materials/Test 1 Definition.pdf]

Test 2: Descriptions

1. Read two descriptions below carefully. Choose one you prefer to use.
2. Write down your answer on the Answer Sheet

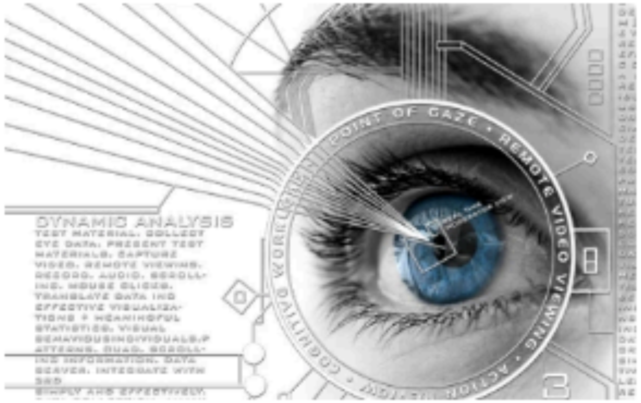

Description 1

1

Eye tracking technology allows a device to know exactly where your eyes are focused. It can indicate your presence, attention, focus, drowsiness, consciousness or other mental states. This information can be used to gain deep insights into consumer behaviour or to design revolutionary new user interfaces across various devices.

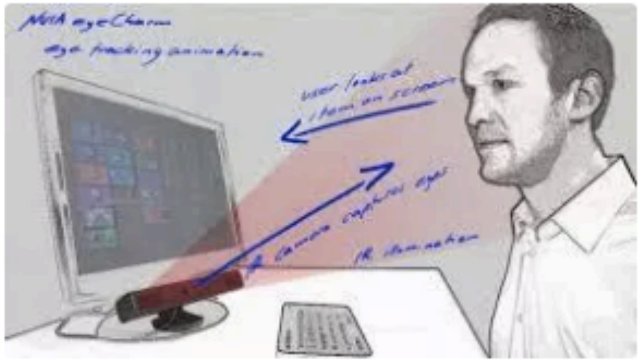

Description 2

2

In the simplest terms, eye tracking is the measurement of eye activity. Where do we look? What do we ignore? When do we blink? How does the pupil react to different stimuli? The concept is basic, but the process and interpretation can be quite complex.
