# Supplementary material for: Eye-tracking technology in identifying visualizers and verbalizers: data on eye-movement differences and detection accuracy
Source: Data Brief. 2019 Aug 29;26:104447. doi: 10.1016/j.dib.2019.104447 (PMC6811880; doi:10.1016/j.dib.2019.104447)
Supplement: Multimedia component 1 [file mmc1.zip › Data Data in Brief/1 Experiment Materials/Test 2 Choose a piece of news.pdf]

More from BBC News

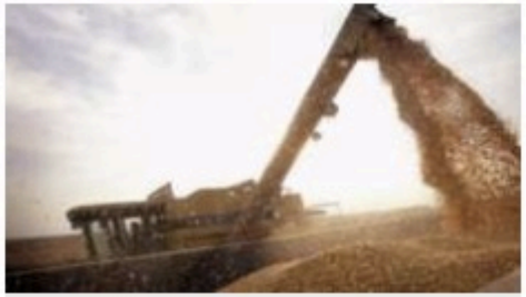

Business

Bayer confirms $66bn Monsanto takeover

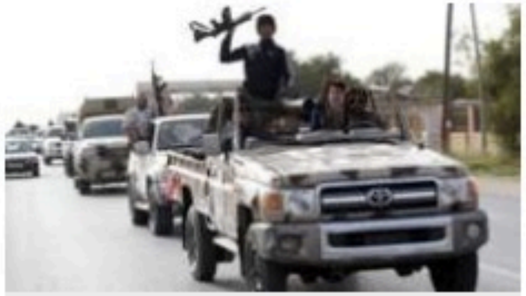

UK Politics

MPs attack Cameron over Libya 'collapse'

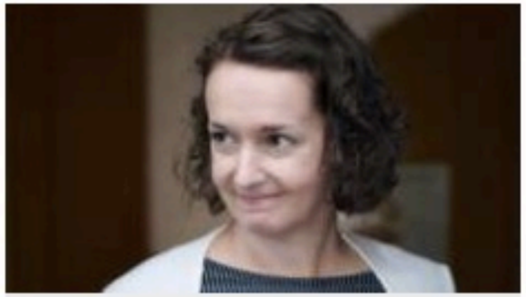

Health

UK Ebola nurse cleared of misconduct

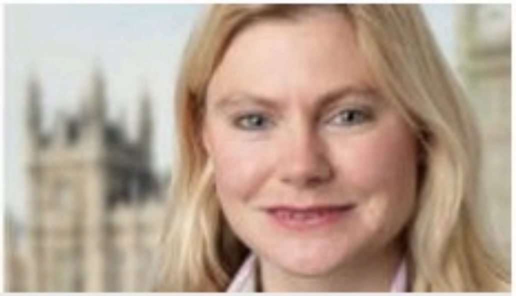

Education & Family

Parent governor role 'won't be scrapped'

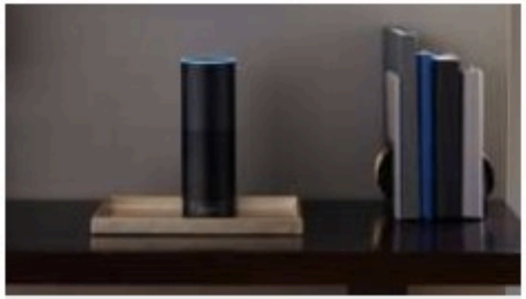

Technology

Amazon brings Echo speakers to UK

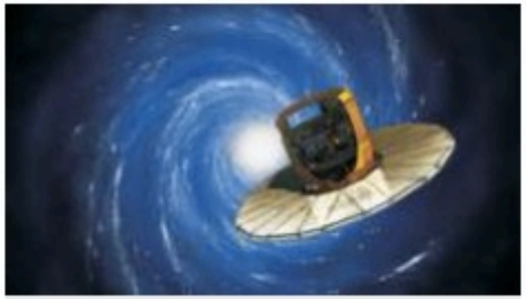

Science & Environment

Celestial mapper plots a billion stars

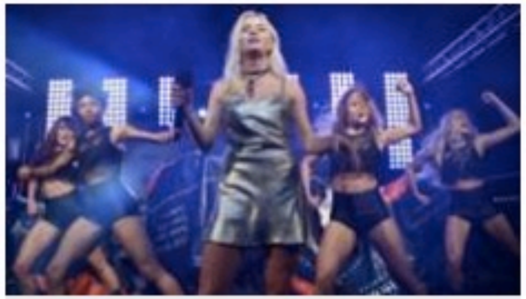

Entertainment & Arts

YouTube ordered to pay more for music

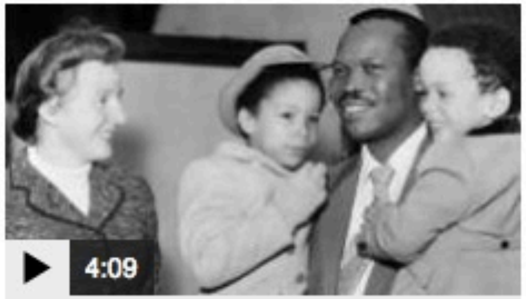

Magazine

The love story that shocked the world

BBC News Services
