# Supplementary material for: Eye-tracking technology in identifying visualizers and verbalizers: data on eye-movement differences and detection accuracy
Source: Data Brief. 2019 Aug 29;26:104447. doi: 10.1016/j.dib.2019.104447 (PMC6811880; doi:10.1016/j.dib.2019.104447)
Supplement: Multimedia component 1 [file mmc1.zip › Data Data in Brief/1 Experiment Materials/Test 3 Reading.pdf]

Reading 1: Deer Populations of the Puget Sound

Nearly any kind of plant of the forest understory can be part of a deer's diet. Where the forest inhibits the growth of grass and other meadow plants, the black-tailed deer browses on huckleberry, salal, dogwood, and almost any other shrub or herb.

Question: The word "inhibits" in the passage is closest in meaning to

A. consists of
B. combines
C. restricts
D. establishes

Reading 2: Groundwater

There it (the underground water) remains, sometimes for long periods, before emerging at the surface again. At first thought it seems incredible that there can be enough space in the "solid" ground underfoot to hold all this water.

Question: The word "incredible" in the passage is closest in meaning to

A. confusing
B. comforting
C. unbelievable
D. interesting

Reading 3: The Origins of Cetaceans

The fossil consists of a complete skull of an archaeocyte, an extinct group of ancestors of modern cetaceans. Although limited to a skull, the Pakicetus fossil provides precious details on the origins of cetaceans. The skull is cetacean-like but its jawbones lack the enlarged space that is filled with fat or oil and used for receiving underwater sound in modern whales. Pakicetus probably detected sound through the ear opening as in land mammals. The skull also lacks a blowhole, another cetacean adaptation for diving. Other features, however, show experts that Pakicetus is a transitional form between a group of extinct flesh-eating mammals, the mesonychids, and cetaceans. It has been suggested that Pakicetus fed on fish in shallow water and was not yet adapted for life in the open ocean. It probably bred and gave birth on land.

Question: The word it in the passage refers to

A. Pakicetus B. Fish C. Life D. Ocean
