# Supplementary material for: Eye-tracking technology in identifying visualizers and verbalizers: data on eye-movement differences and detection accuracy
Source: Data Brief. 2019 Aug 29;26:104447. doi: 10.1016/j.dib.2019.104447 (PMC6811880; doi:10.1016/j.dib.2019.104447)
Supplement: Multimedia component 1 [file mmc1.zip › Data Data in Brief/1 Experiment Materials/Test 4 pictures.pdf]

Mark out the part you like in each picture

4.1.

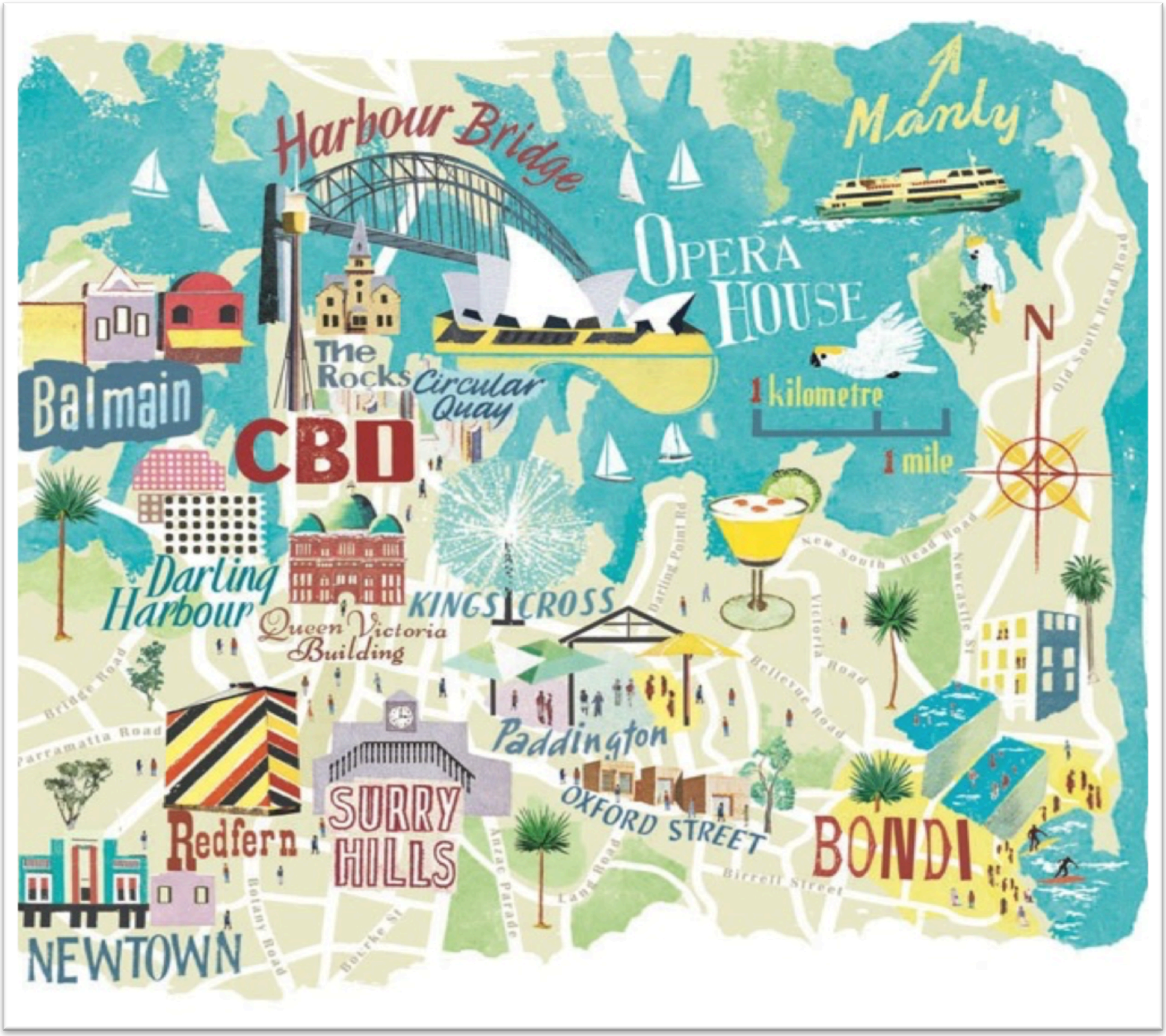
Harbour Bridge
Manly
Opera House
The Rocks
Circular Quay
Balmain
CBD
1 kilometre
1 mile
N
Old South Head Road
New South Head Road
Newcastle St
Darling Point Rd
Victoria Road
Bellevue Road
Darling Harbour
Queen Victoria Building
Kings Cross
Bridge Road
Parramatta Road
Paddington
Oxford Street
Surry Hills
Redfern
Bondi
Birrell Street
Lang Road
Anzac Parade
Botany Road
Bourke St
Newtown

4.2.

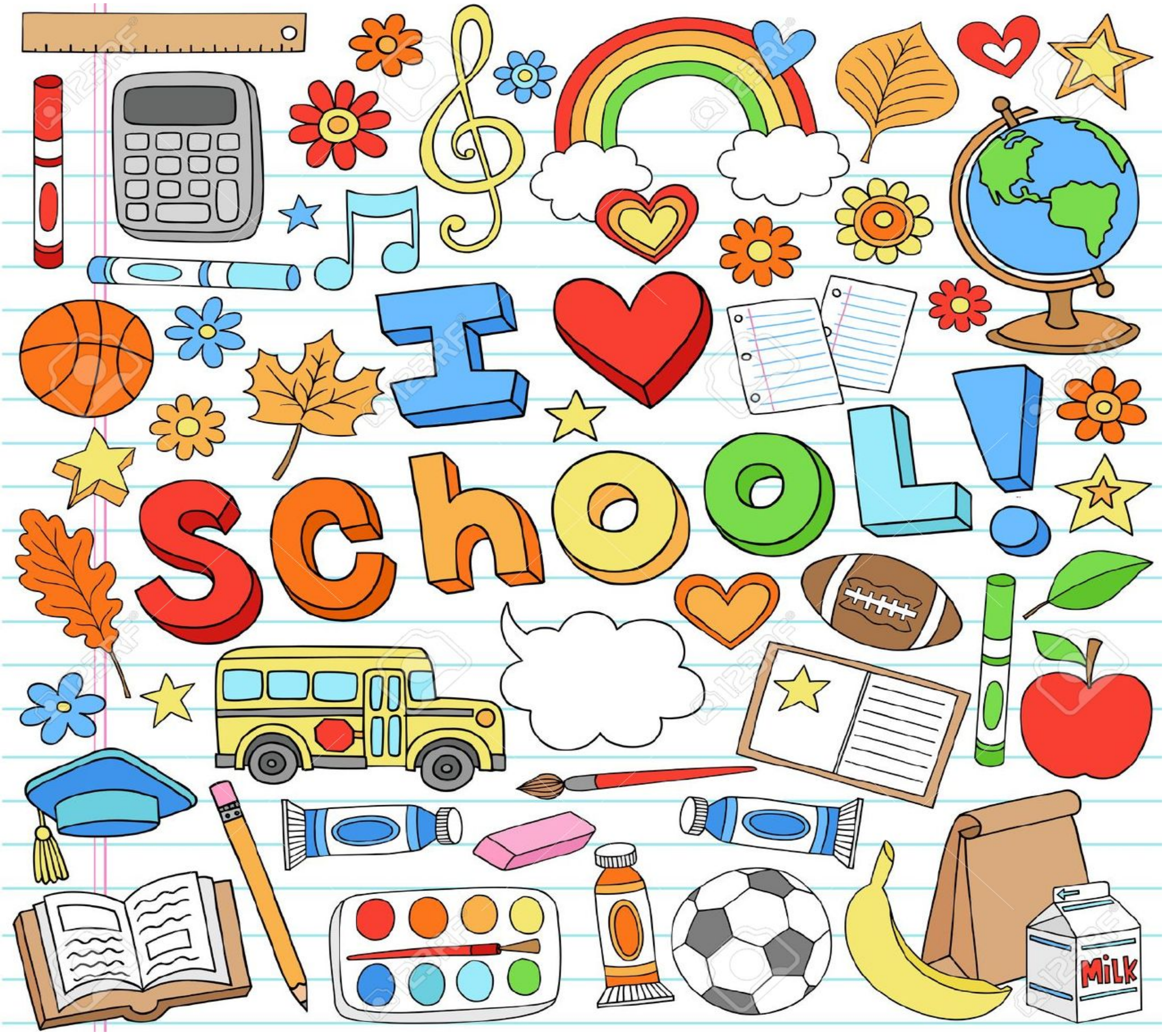

4.3.

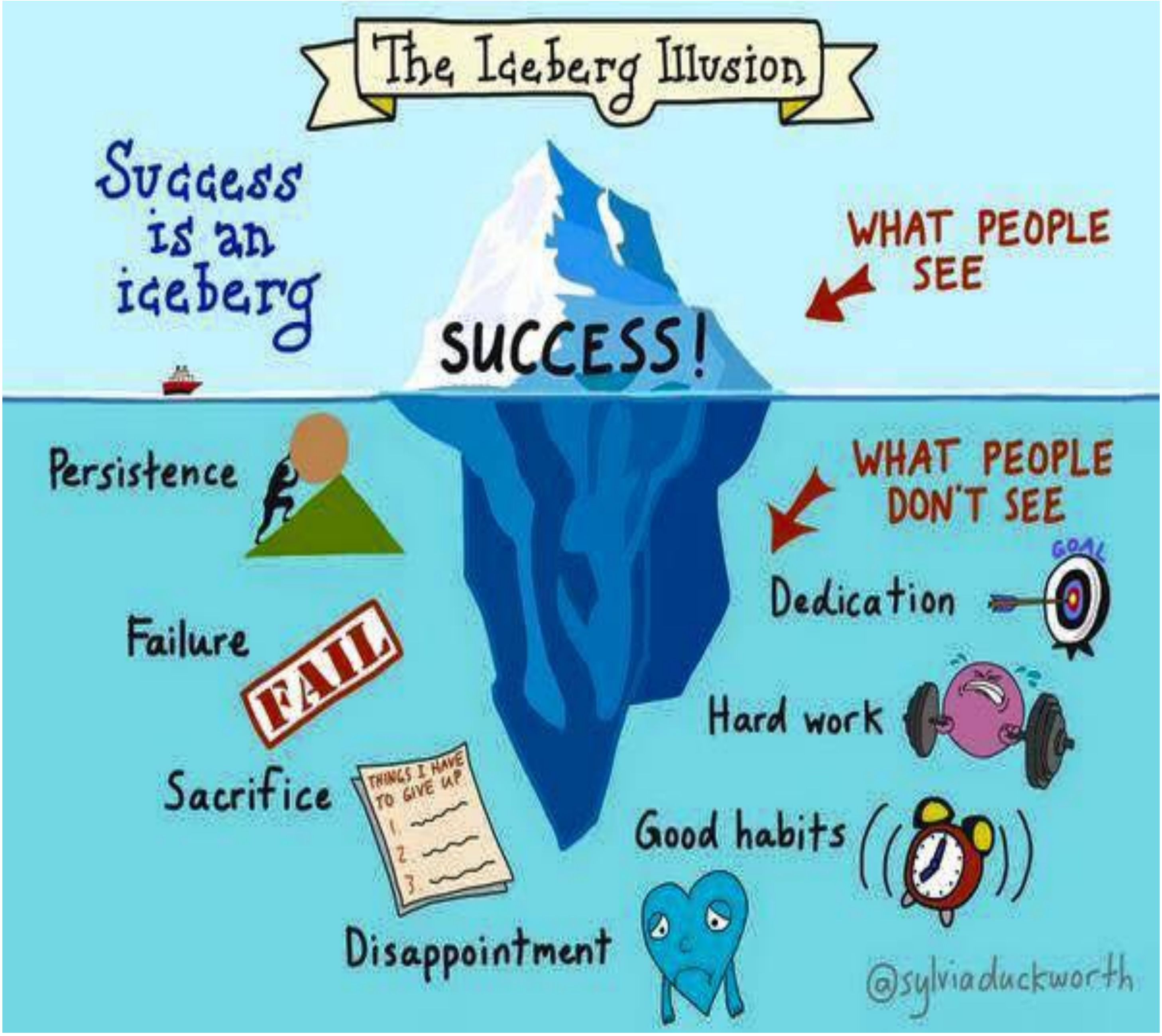

The Iceberg Illusion
Success is an iceberg
WHAT PEOPLE SEE
SUCCESS!
WHAT PEOPLE DON'T SEE
Persistence
Dedication
GOAL
Failure
FAIL
Hard work
Sacrifice
THINGS I HAVE TO GIVE UP
1.
2.
3.
Good habits
Disappointment
@sylviaduckworth

4.4.

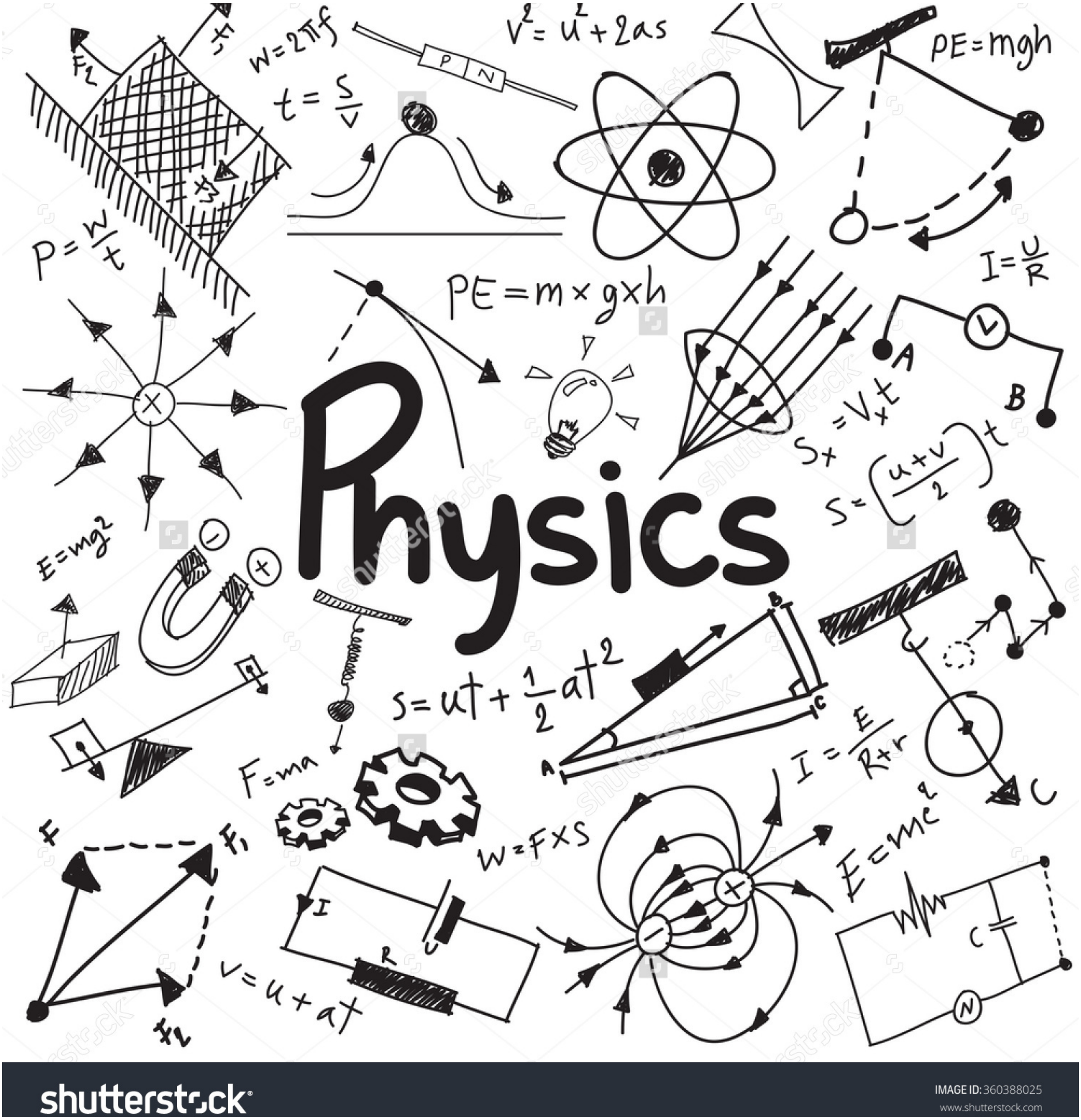
Physics
W=2πf
v²=u²+2as
PE=mgh
t=S/V
P N
P=W/t
PE=m×g×h
I=U/R
A
B
S=(u+v/2)t
E=mg²
s=ut+½at²
F=ma
I=E/R+r
W=F×S
E=mc²
v=u+at
F
F₁
F₂
I
R
U
C
N
shutterstock
IMAGE ID: 360388025
www.shutterstock.com

4.5.

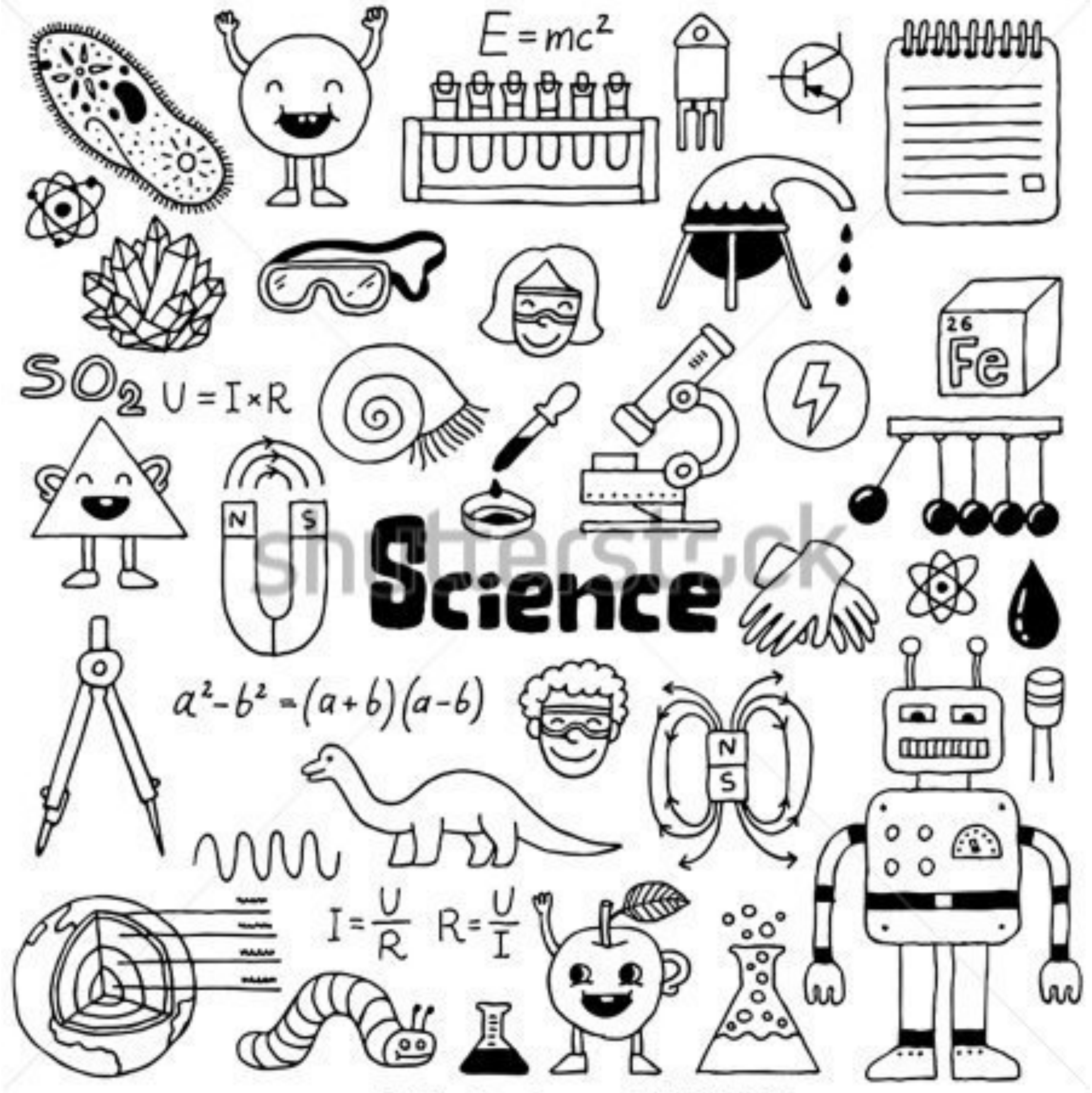

www.shutterstock.com · 197415662

4.6.

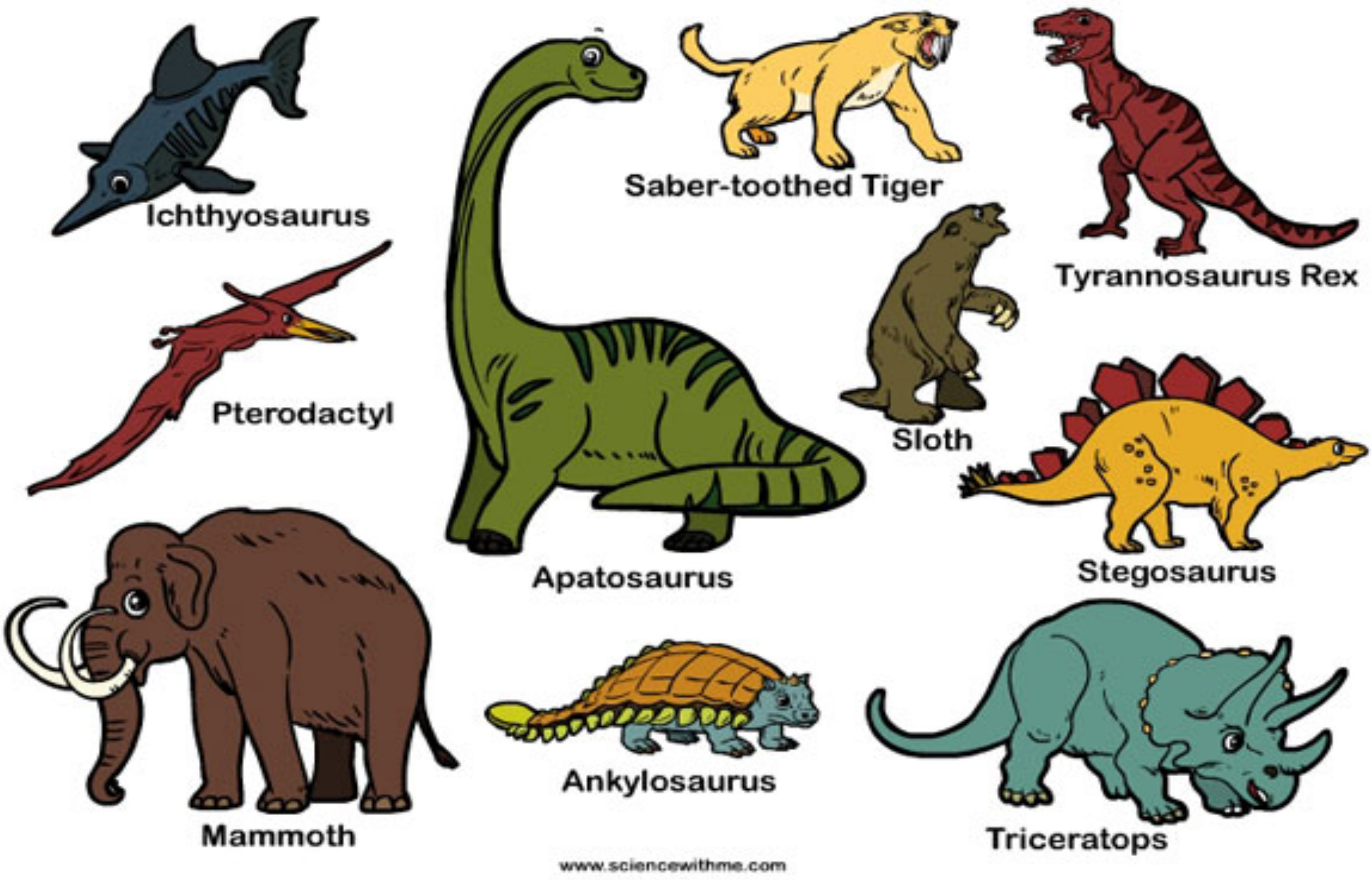
Ichthyosaurus
Saber-toothed Tiger
Tyrannosaurus Rex
Pterodactyl
Sloth
Apatosaurus
Stegosaurus
Mammoth
Ankylosaurus
Triceratops
www.sciencewithme.com
