# Supplementary material for: Eye-tracking technology in identifying visualizers and verbalizers: data on eye-movement differences and detection accuracy
Source: Data Brief. 2019 Aug 29;26:104447. doi: 10.1016/j.dib.2019.104447 (PMC6811880; doi:10.1016/j.dib.2019.104447)
Supplement: Multimedia component 1 [file mmc1.zip › Data Data in Brief/2 Forms- ethics, answer sheet, ILS scoring sheet and ILS questionnaire/Document 1 Answer Sheet .docx]

**Answer Sheet for the Experiment**

Date:___________________

Time of the test:___________________

Test 1:

Please choose one description you like.

Your Choice:

A. Description 1 B. Description 2

Test 2

Choose two pieces of news you are interested in, and explain why.

News 1: ­­­______________________ Reason: ________________________________________________________

News 2: ­­­______________________ Reason: ________________________________________________________

Test 3

View the website and answer the questions.

1. After reading the material, what impress you most? Please give several key words.

__________________

__________________

__________________

1. What do you want to share with us, after reading the material?

___________________________________________________________________________________________________________________________________________________________________________________________________________________________________________________________________________________________________________________________________________________________________________________________________________________________________________________________________________________________________________________________________________________________________________

Test 4

1. There are 5 pictures. Watch each one for 10 seconds.
2. Mark out the part you like in each picture. You can write or draw.

| Example 1: | 4.1. | 4.2. | 4.3. |
| --- | --- | --- | --- |
| 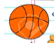  basketball |  |  |  |
|  | 4.4. | 4.5. | 4.6. |
|  |  |  |  |
