# Supplementary material for: Eye-tracking technology in identifying visualizers and verbalizers: data on eye-movement differences and detection accuracy
Source: Data Brief. 2019 Aug 29;26:104447. doi: 10.1016/j.dib.2019.104447 (PMC6811880; doi:10.1016/j.dib.2019.104447)
Supplement: Multimedia component 1 [file mmc1.zip › Data Data in Brief/2 Forms- ethics, answer sheet, ILS scoring sheet and ILS questionnaire/Document 2 ILS Scoring Sheet.docx]

**Learning Styles Questionnaire Scoring Sheet**

1. Place a “1” in the appropriate spaces in the table below (e.g. if you answered "a" to Question 3, put a "1" in Column "a" by Question 3).
2. Add up the columns and write the totals in the indicated spaces.

| **Activist/Reflector** | | | **Sensing/Intuitive** | | | **Visual/Textual** | | | **Sequential/Global** | | |
| --- | --- | --- | --- | --- | --- | --- | --- | --- | --- | --- | --- |
| Q | a | b | Q | a | b | Q | a | b | Q | a | b |
| 1 |  |  | 2 |  |  | 3 |  |  | 4 |  |  |
| 5 |  |  | 6 |  |  | 7 |  |  | 8 |  |  |
| 9 |  |  | 10 |  |  | 11 |  |  | 12 |  |  |
| 13 |  |  | 14 |  |  | 15 |  |  | 16 |  |  |
| 17 |  |  | 18 |  |  | 19 |  |  | 20 |  |  |
| 21 |  |  | 22 |  |  | 23 |  |  | 24 |  |  |
| 25 |  |  | 26 |  |  | 27 |  |  | 28 |  |  |
| 29 |  |  | 30 |  |  | 31 |  |  | 32 |  |  |
| 33 |  |  | 34 |  |  | 35 |  |  | 36 |  |  |
| 37 |  |  | 38 |  |  | 39 |  |  | 40 |  |  |
| 41 |  |  | 42 |  |  | 43 |  |  | 44 |  |  |
| *Total (add up each colume)* | | | | | | | | | | | |
| **Activist/Reflector** | | | **Sensing/Intuitive** | | | **Visual/Textual** | | | **Sequential/Global** | | |
| Q | a | b | Q | a | b | Q | a | b | Q | a | b |
| N/A |  |  | N/A |  |  | N/A |  |  | N/A |  |  |
