# Supplementary material for: Eye-tracking technology in identifying visualizers and verbalizers: data on eye-movement differences and detection accuracy
Source: Data Brief. 2019 Aug 29;26:104447. doi: 10.1016/j.dib.2019.104447 (PMC6811880; doi:10.1016/j.dib.2019.104447)
Supplement: Multimedia component 1 [file mmc1.zip › Data Data in Brief/2 Forms- ethics, answer sheet, ILS scoring sheet and ILS questionnaire/Document 3 ILS questionnaire.docx]

## Index of Learning Styles Questionnaire

1. I understand something better after I

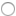
    **(a)** try it out.

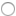
    **(b)** think it through.
2. I would rather be considered

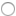
    **(a)** realistic.

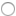
    **(b)** innovative.
3. When I think about what I did yesterday, I am most likely to get

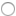
    **(a)** a picture.

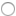
    **(b)** words.
4. I tend to

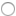
    **(a)** understand details of a subject but may be fuzzy about its overall structure.

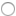
    **(b)** understand the overall structure but may be fuzzy about details.
5. When I am learning something new, it helps me to

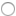
    **(a)** talk about it.

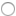
    **(b)** think about it.
6. If I were a teacher, I would rather teach a course

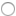
    **(a)** that deals with facts and real life situations.

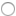
    **(b)** that deals with ideas and theories.
7. I prefer to get new information in

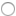
    **(a)** pictures, diagrams, graphs, or maps.

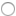
    **(b)** written directions.
8. Once I understand

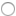
    **(a)** all the parts, I understand the whole thing.

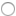
    **(b)** the whole thing, I see how the parts fit.
9. In a study group working on difficult material, I am more likely to

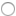
    **(a)** jump in and contribute ideas.

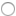
    **(b)** sit back and listen.
10. I find it easier

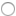
    **(a)** to learn facts.

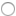
    **(b)** to learn concepts.
11. In a book with lots of pictures and charts, I am likely to

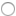
    **(a)** look over the pictures and charts carefully.

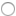
    **(b)** focus on the written text.
12. When I solve math problems

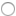
    **(a)** I usually work my way to the solutions one step at a time.

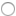
    **(b)** I often just see the solutions but then have to struggle to figure out the steps to get to them.
13. In classes I have taken

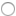
    **(a)** I have usually gotten to know many of the students.

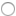
    **(b)** I have rarely gotten to know many of the students.
14. In reading nonfiction, I prefer

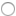
    **(a)** something that teaches me new facts or tells me how to do something.

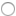
    **(b)** something that gives me new ideas to think about.
15. I like teachers

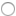
    **(a)** who put a lot of diagrams on the board.

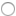
    **(b)** who spend a lot of time explaining.
16. When I'm analyzing a story or a novel

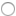
    **(a)** I think of the incidents and try to put them together to figure out the themes.

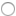
    **(b)** I just know what the themes are when I finish reading and then I have to go back and find the incidents that demonstrate them.
17. When I start a homework problem, I am more likely to

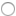
    **(a)** start working on the solution immediately.

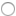
    **(b)** try to fully understand the problem first.
18. I prefer the idea of

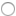
    **(a)** certainty.

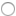
    **(b)** theory.
19. I remember best

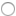
    **(a)** what I see (pictures).

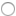
    **(b)** what I hear (words).
20. It is more important to me that an instructor

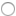
    **(a)** lay out the material in clear sequential steps.

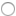
    **(b)** give me an overall picture and relate the material to other subjects.
21. I prefer to study

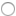
    **(a)** in a study group.

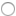
    **(b)** alone.
22. I am more likely to be considered

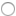
    **(a)** careful about the details of my work.

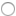
    **(b)** creative about how to do my work.
23. When I get directions to a new place, I prefer

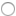
    **(a)** a map.

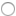
    **(b)** written instructions.
24. I learn

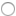
    **(a)** at a fairly regular pace. If I study hard, I'll "get it."

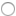
    **(b)** in fits and starts. I'll be totally confused and then suddenly it all "clicks."
25. I would rather first

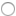
    **(a)** try things out.

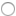
    **(b)** think about how I'm going to do it.
26. When I am reading for enjoyment, I like writers to

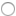
    **(a)** clearly say what they mean.

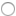
    **(b)** say things in creative, interesting ways.
27. When I see a diagram or sketch in class, I am most likely to remember

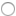
    **(a)** the picture.

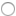
    **(b)** the written explanation.
28. When considering a body of information, I am more likely to

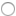
    **(a)** focus on details and miss the big picture.

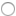
    **(b)** try to understand the big picture before getting into the details.
29. I more easily remember

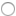
    **(a)** something I have done.

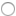
    **(b)** something I have thought a lot about.
30. When I have to perform a task, I prefer to

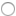
    **(a)** master one way of doing it.

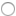
    **(b)** come up with new ways of doing it.
31. When someone is showing me data, I prefer

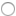
    **(a)** charts or graphs.

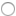
    **(b)** text summarizing the results.
32. When writing a paper, I am more likely to

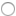
    **(a)** work on (think about or write) the beginning of the paper and progress forward.

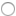
    **(b)** work on (think about or write) different parts of the paper and then order them.
33. When I have to work on a group project, I first want to

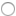
    **(a)** have "group brainstorming" where everyone contributes ideas.

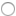
    **(b)** brainstorm individually and then come together as a group to compare ideas.
34. I consider it higher praise to call someone

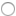
    **(a)** sensible.

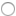
    **(b)** imaginative.
35. When I meet people at a party, I am more likely to remember

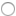
    **(a)** what they looked like.

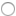
    **(b)** what they said about themselves.
36. When I am learning a new subject, I prefer to

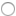
    **(a)** stay focused on that subject, learning as much about it as I can.

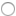
    **(b)** try to make connections between that subject and related subjects.
37. I am more likely to be considered

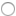
    **(a)** outgoing.

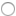
    **(b)** reserved.
38. I prefer courses that emphasize

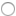
    **(a)** concrete material (facts, data).

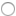
    **(b)** abstract material (concepts, theories).
39. For entertainment, I would rather

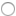
    **(a)** watch television.

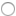
    **(b)** read a book.
40. Some teachers start their lectures with an outline of what they will cover. Such outlines are

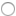
    **(a)** somewhat helpful to me.

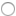
    **(b)** very helpful to me.
41. The idea of doing homework in groups, with one grade for the entire group,

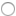
    **(a)** appeals to me.

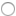
    **(b)** does not appeal to me.
42. When I am doing long calculations,

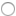
    **(a)** I tend to repeat all my steps and check my work carefully.

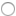
    **(b)** I find checking my work tiresome and have to force myself to do it.
43. I tend to picture places I have been

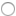
    **(a)** easily and fairly accurately.

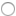
    **(b)** with difficulty and without much detail.
44. When solving problems in a group, I would be more likely to

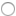
    **(a)** think of the steps in the solution process.

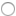
    **(b)** think of possible consequences or applications of the solution in a wide range of areas.
