# Supplementary figures and images for: Eye-tracking technology in identifying visualizers and verbalizers: data on eye-movement differences and detection accuracy
Source: Data Brief. 2019 Aug 29;26:104447. doi: 10.1016/j.dib.2019.104447 (PMC6811880; doi:10.1016/j.dib.2019.104447)

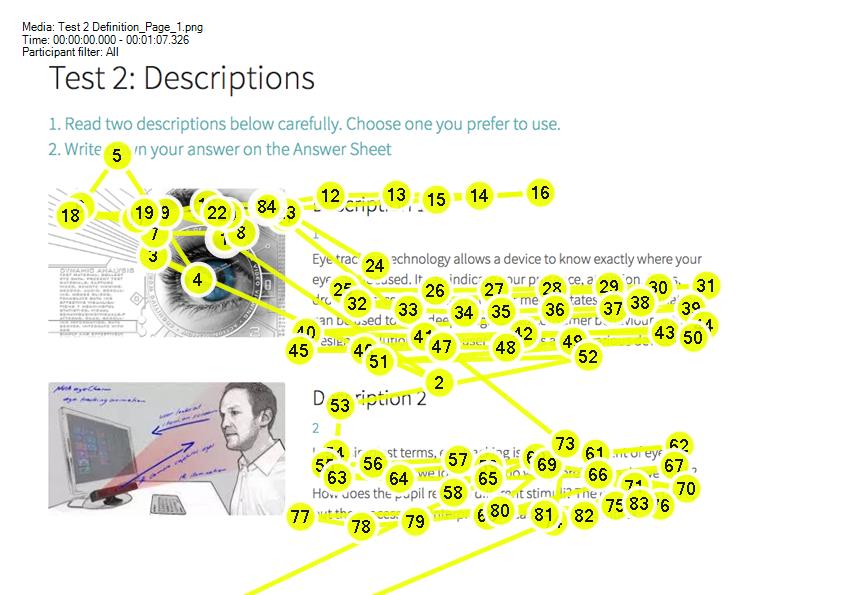

Supplement: Multimedia component 1 [file mmc1.zip › Data Data in Brief/4 Visual Data- Splited/Document 1/definition rec01.jpg]

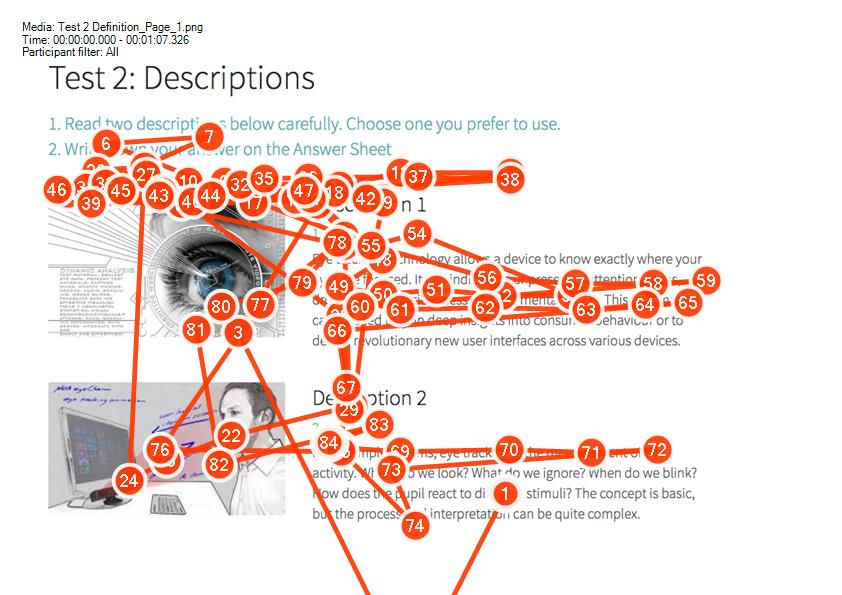

Supplement: Multimedia component 1 [file mmc1.zip › Data Data in Brief/4 Visual Data- Splited/Document 1/definition rec02.jpg]

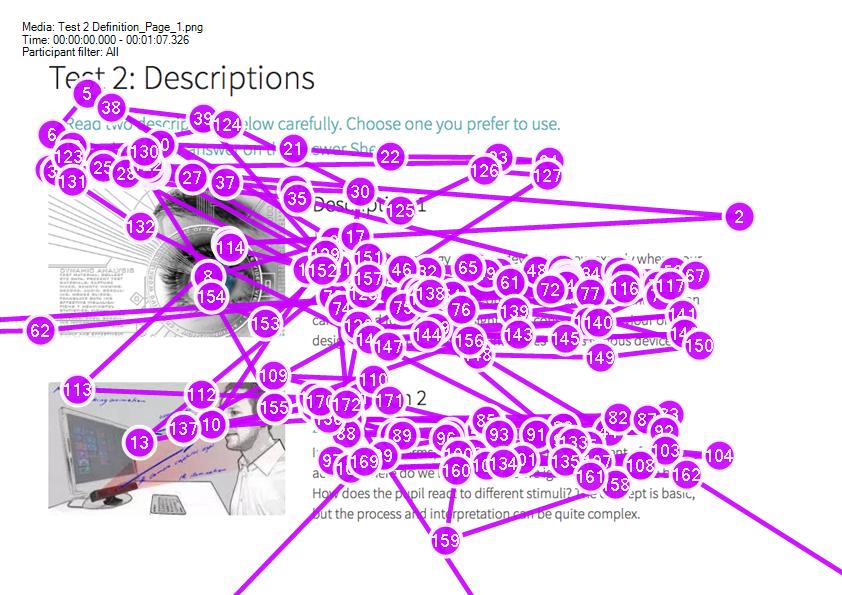

Supplement: Multimedia component 1 [file mmc1.zip › Data Data in Brief/4 Visual Data- Splited/Document 1/definition rec03.jpg]

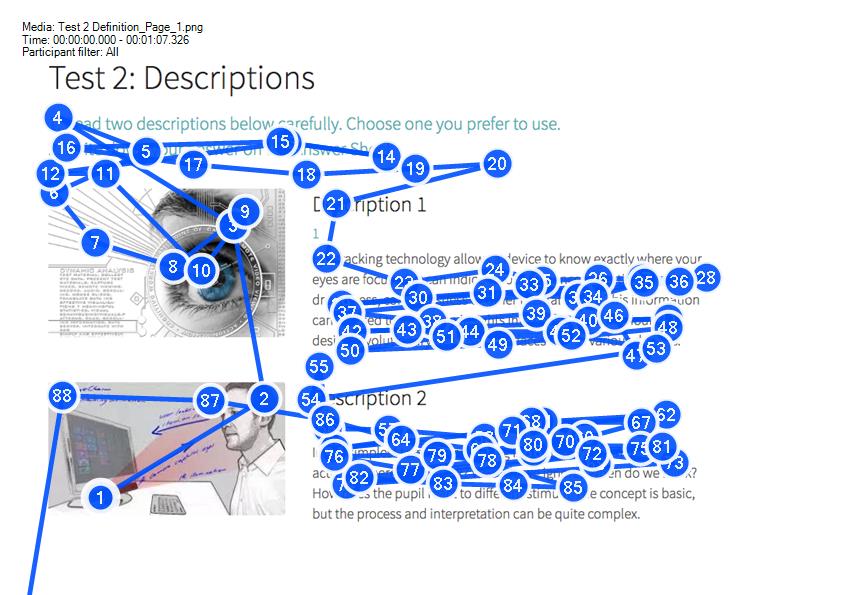

Supplement: Multimedia component 1 [file mmc1.zip › Data Data in Brief/4 Visual Data- Splited/Document 1/definition rec04.jpg]

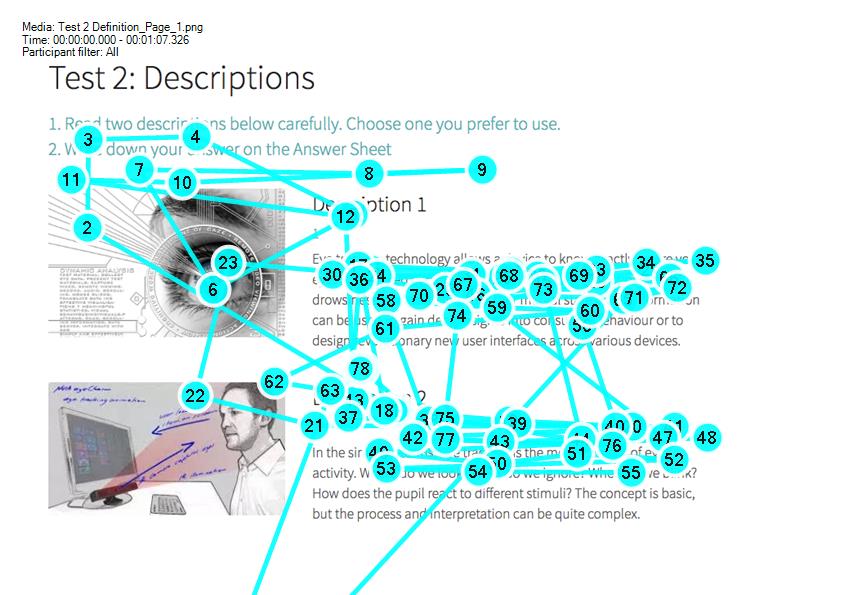

Supplement: Multimedia component 1 [file mmc1.zip › Data Data in Brief/4 Visual Data- Splited/Document 1/definition rec05.jpg]

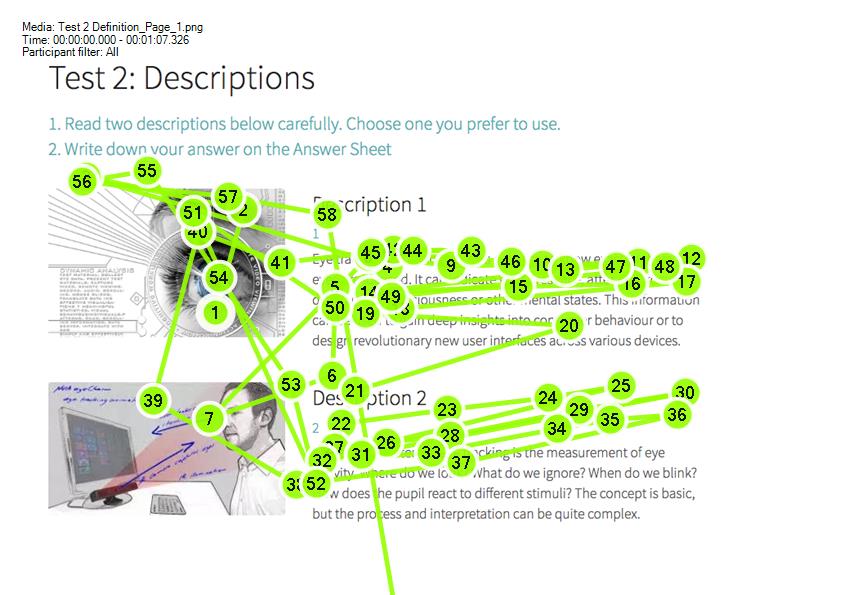

Supplement: Multimedia component 1 [file mmc1.zip › Data Data in Brief/4 Visual Data- Splited/Document 1/definition rec06.jpg]

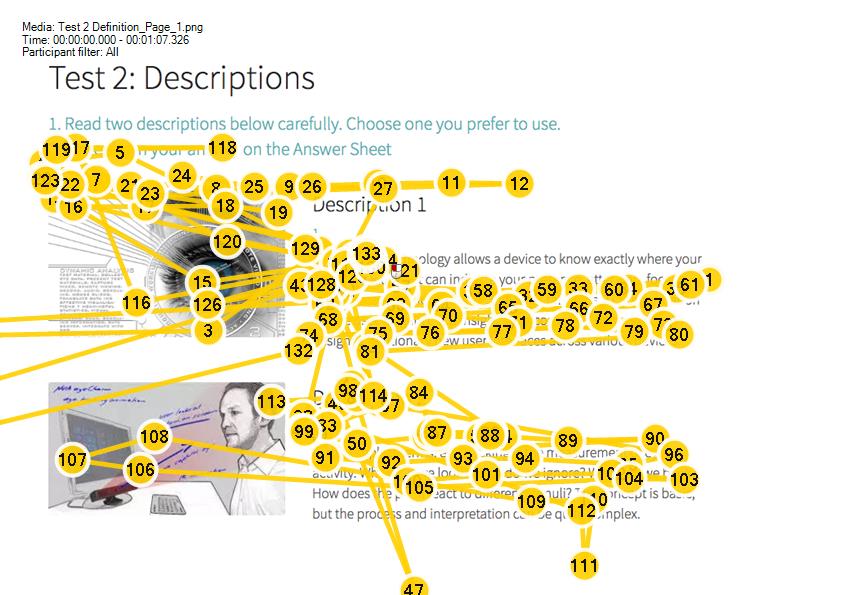

Supplement: Multimedia component 1 [file mmc1.zip › Data Data in Brief/4 Visual Data- Splited/Document 1/definition rec07.jpg]

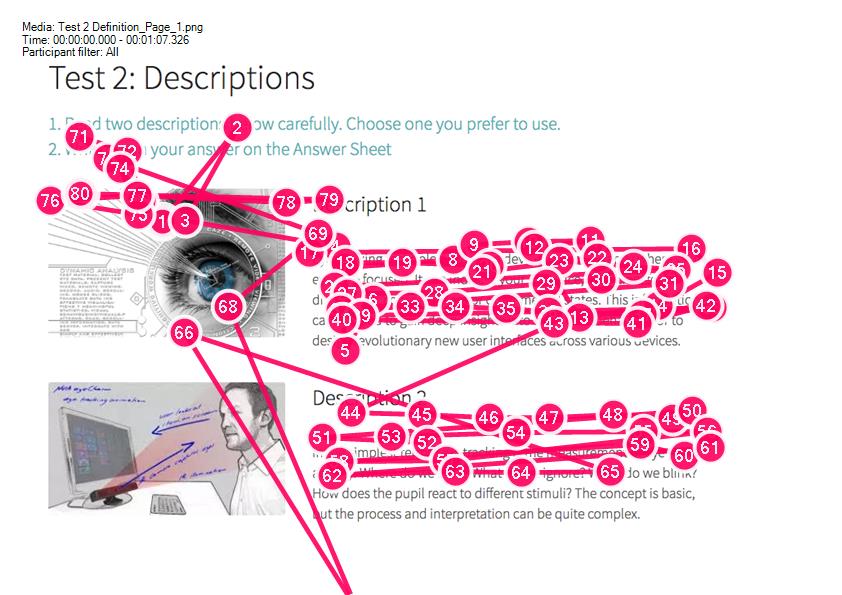

Supplement: Multimedia component 1 [file mmc1.zip › Data Data in Brief/4 Visual Data- Splited/Document 1/definition rec08.jpg]

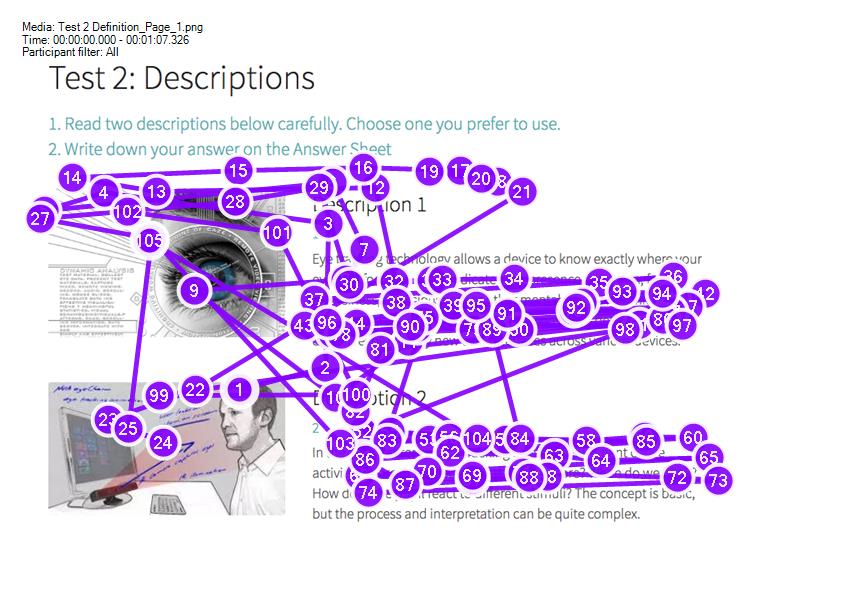

Supplement: Multimedia component 1 [file mmc1.zip › Data Data in Brief/4 Visual Data- Splited/Document 1/definition rec09.jpg]

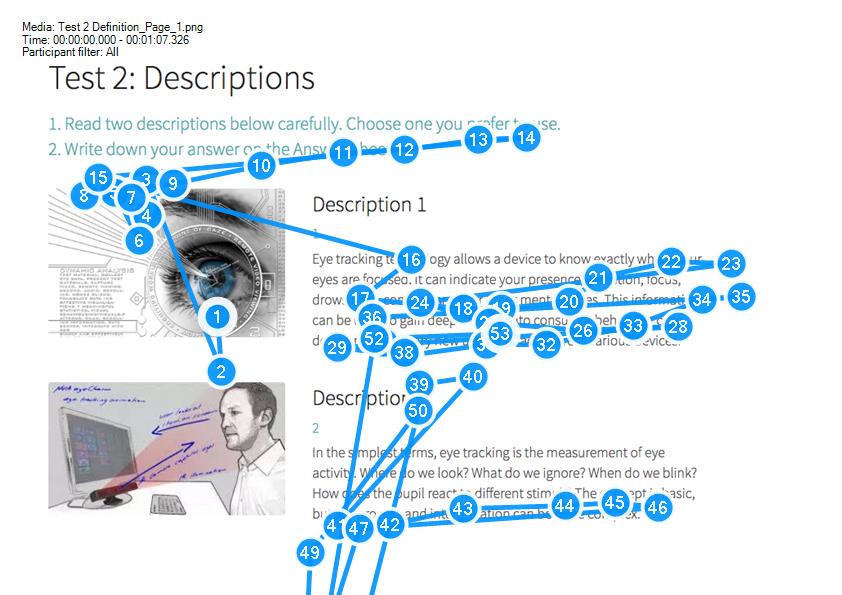

Supplement: Multimedia component 1 [file mmc1.zip › Data Data in Brief/4 Visual Data- Splited/Document 1/definition rec10.jpg]

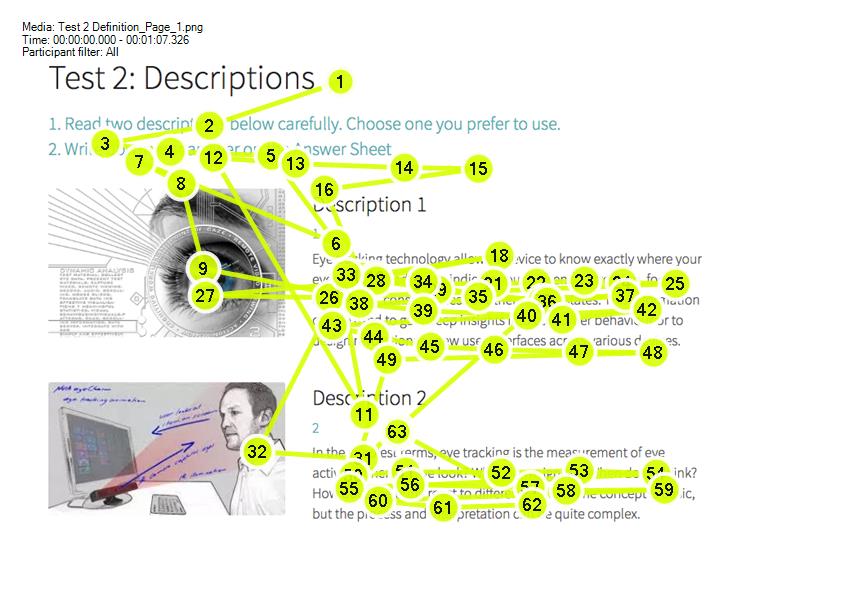

Supplement: Multimedia component 1 [file mmc1.zip › Data Data in Brief/4 Visual Data- Splited/Document 1/definition rec11.jpg]

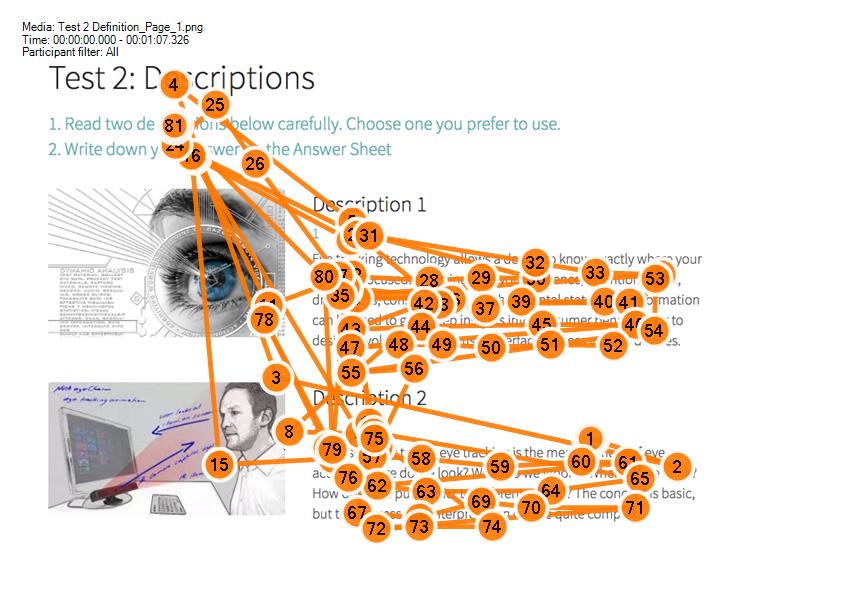

Supplement: Multimedia component 1 [file mmc1.zip › Data Data in Brief/4 Visual Data- Splited/Document 1/definition rec12.jpg]

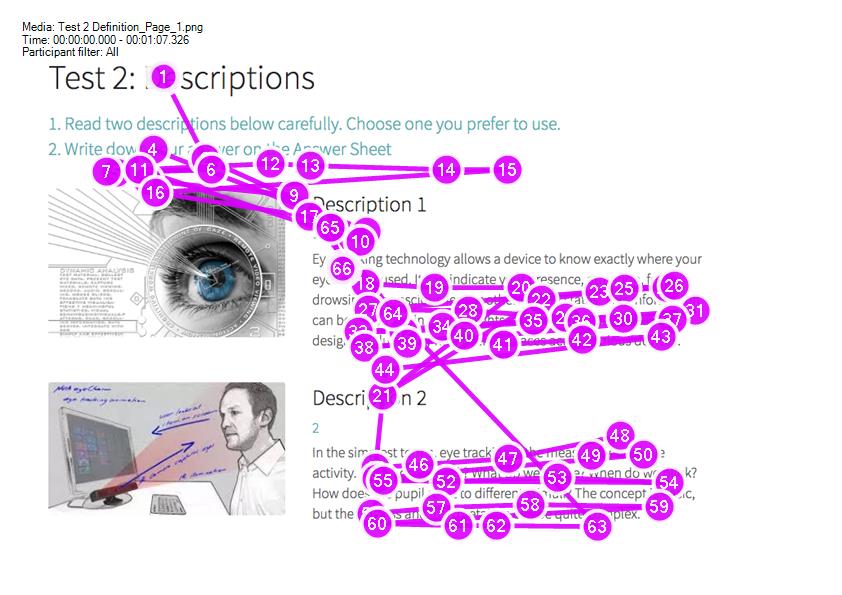

Supplement: Multimedia component 1 [file mmc1.zip › Data Data in Brief/4 Visual Data- Splited/Document 1/definition rec13.jpg]

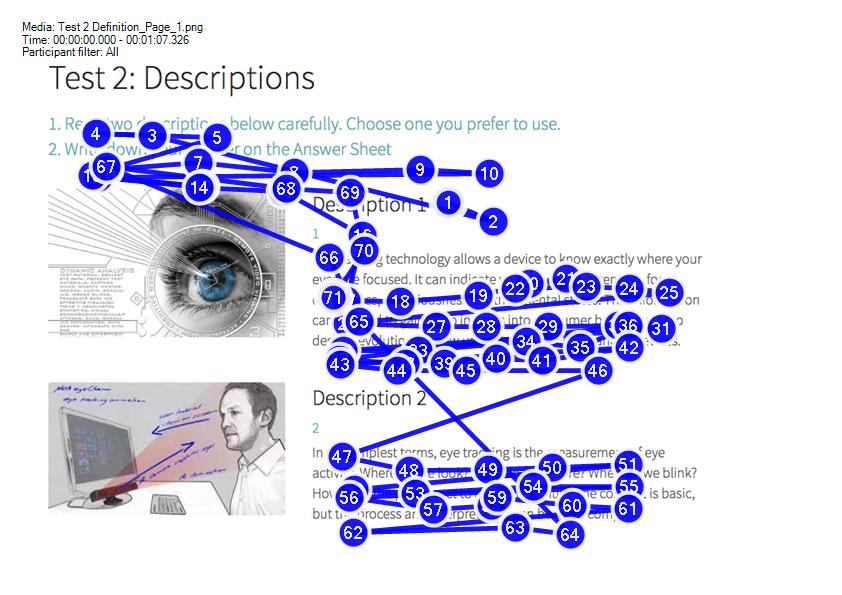

Supplement: Multimedia component 1 [file mmc1.zip › Data Data in Brief/4 Visual Data- Splited/Document 1/definition rec14.jpg]

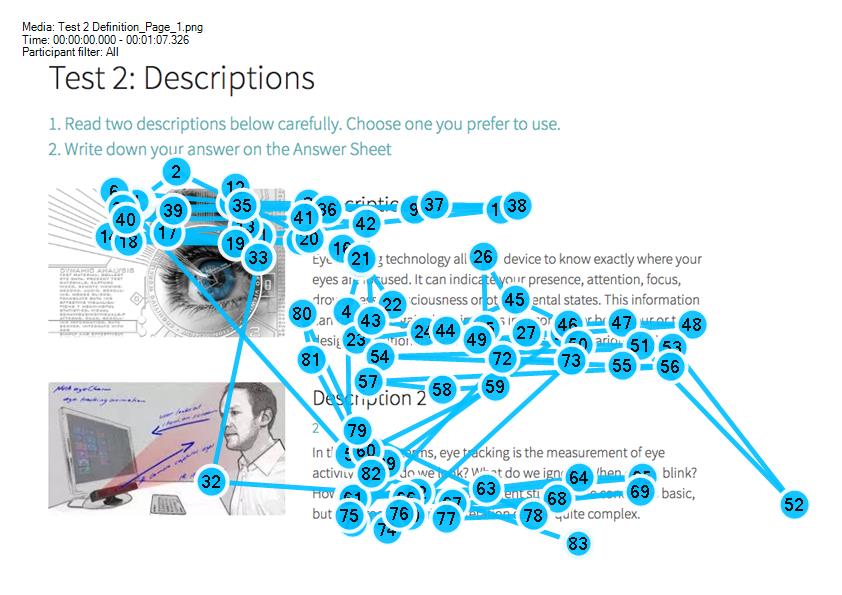

Supplement: Multimedia component 1 [file mmc1.zip › Data Data in Brief/4 Visual Data- Splited/Document 1/definition rec15.jpg]

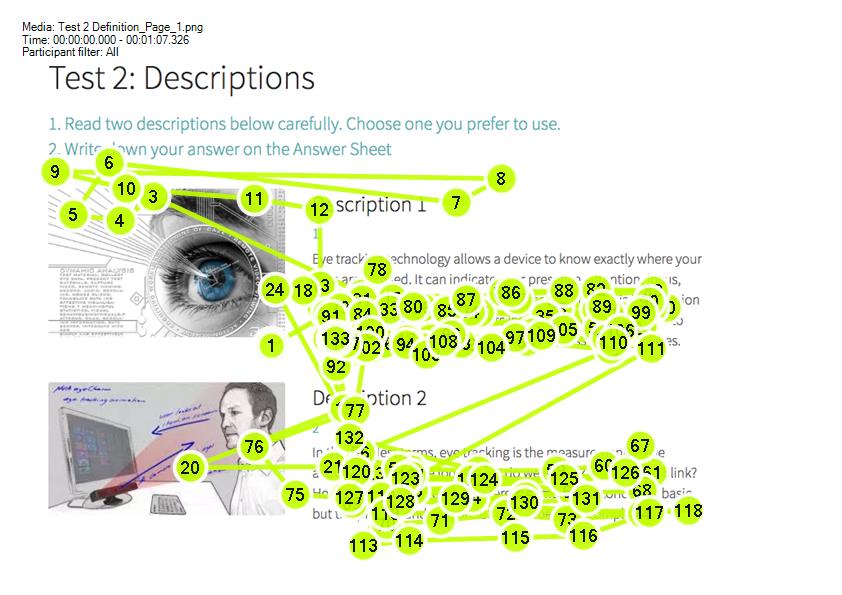

Supplement: Multimedia component 1 [file mmc1.zip › Data Data in Brief/4 Visual Data- Splited/Document 1/definition rec16.jpg]

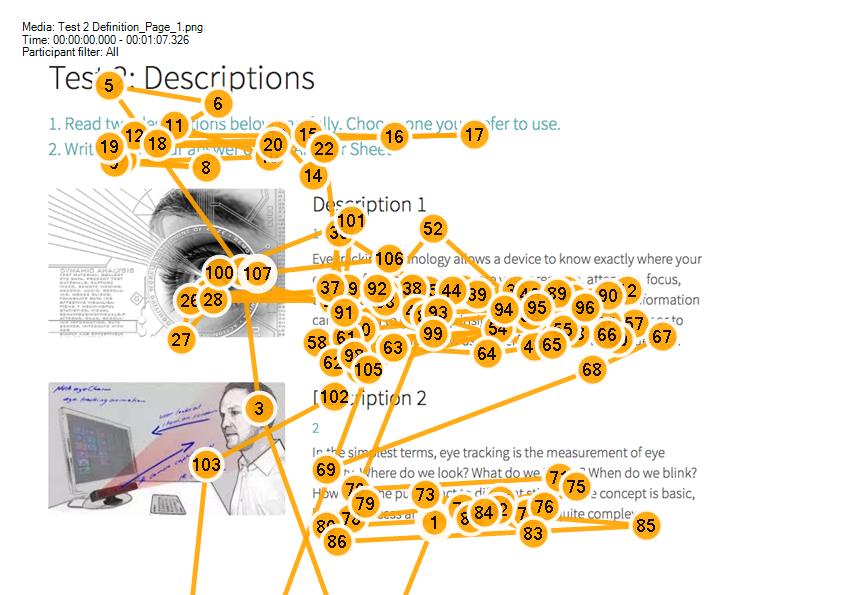

Supplement: Multimedia component 1 [file mmc1.zip › Data Data in Brief/4 Visual Data- Splited/Document 1/definition rec17.jpg]

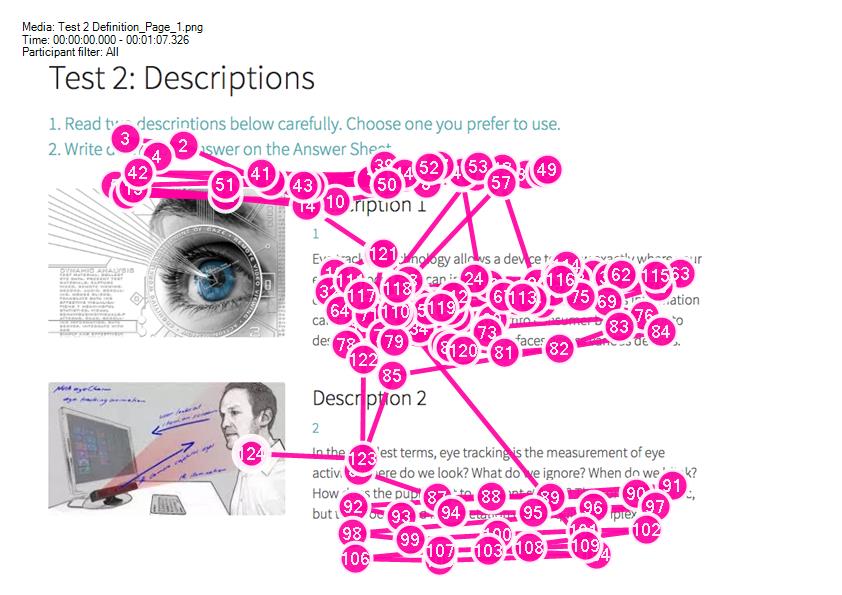

Supplement: Multimedia component 1 [file mmc1.zip › Data Data in Brief/4 Visual Data- Splited/Document 1/definition rec18.jpg]

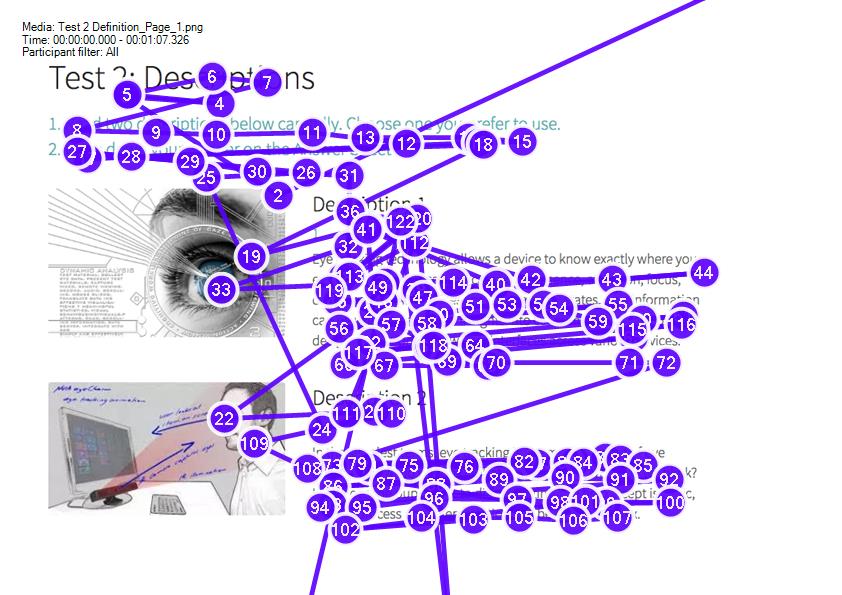

Supplement: Multimedia component 1 [file mmc1.zip › Data Data in Brief/4 Visual Data- Splited/Document 1/definition rec19.jpg]

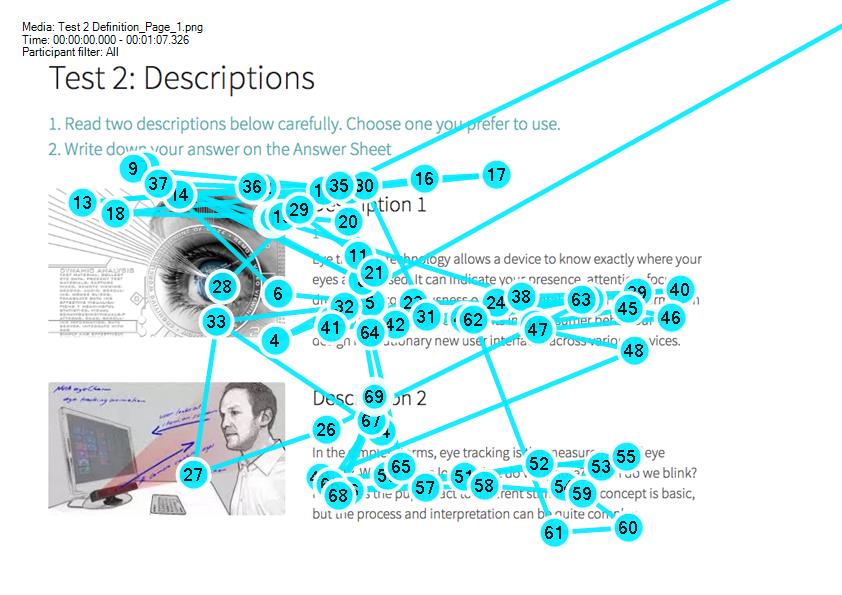

Supplement: Multimedia component 1 [file mmc1.zip › Data Data in Brief/4 Visual Data- Splited/Document 1/definition rec20.jpg]

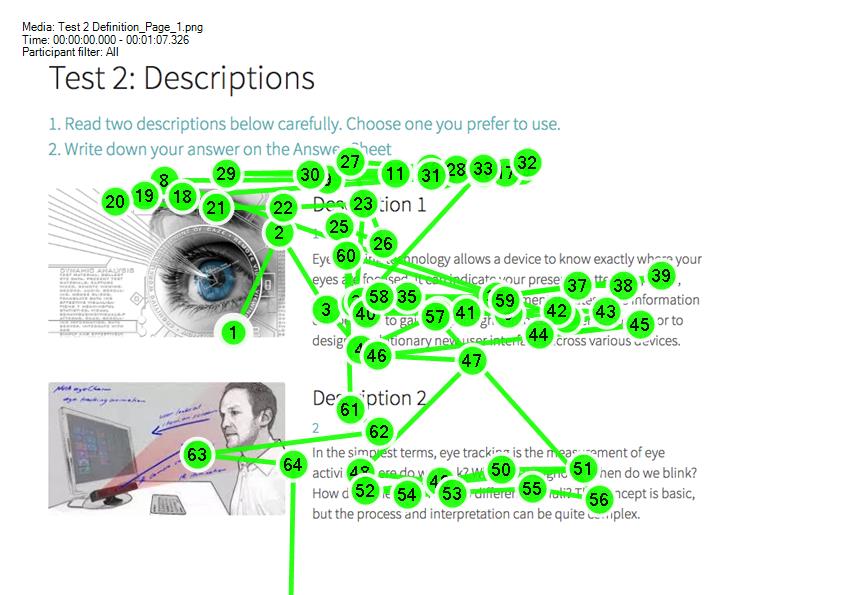

Supplement: Multimedia component 1 [file mmc1.zip › Data Data in Brief/4 Visual Data- Splited/Document 1/definition rec21.jpg]

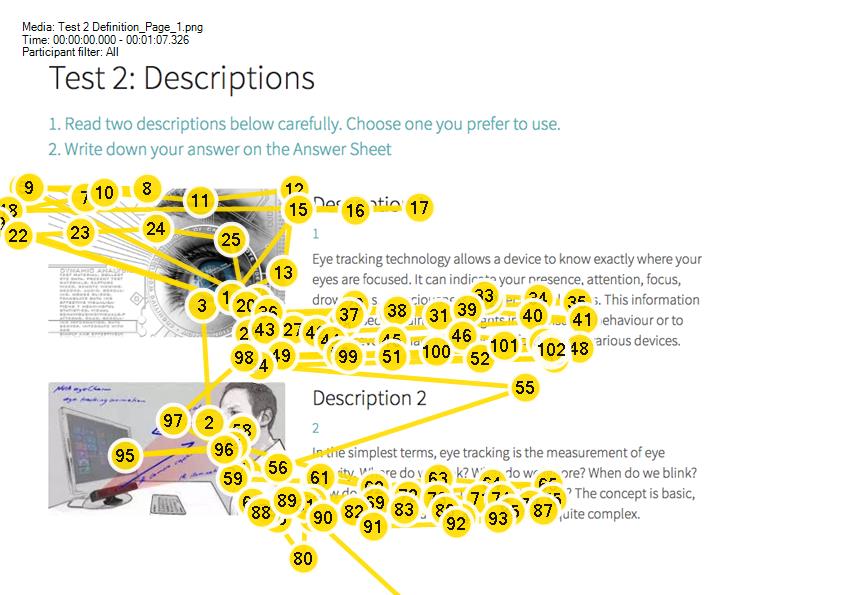

Supplement: Multimedia component 1 [file mmc1.zip › Data Data in Brief/4 Visual Data- Splited/Document 1/definition rec22.jpg]

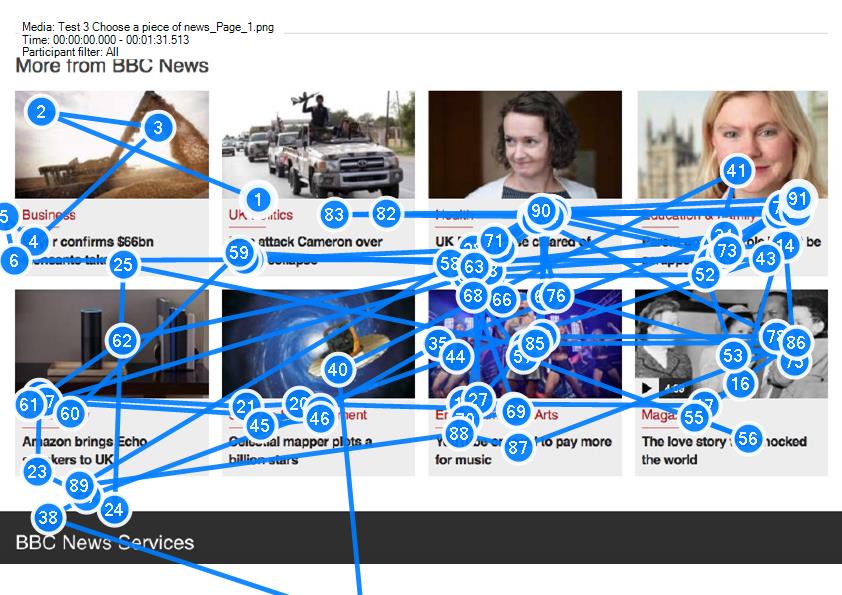

Supplement: Multimedia component 1 [file mmc1.zip › Data Data in Brief/4 Visual Data- Splited/Document 2/3 rec pilot.jpg]

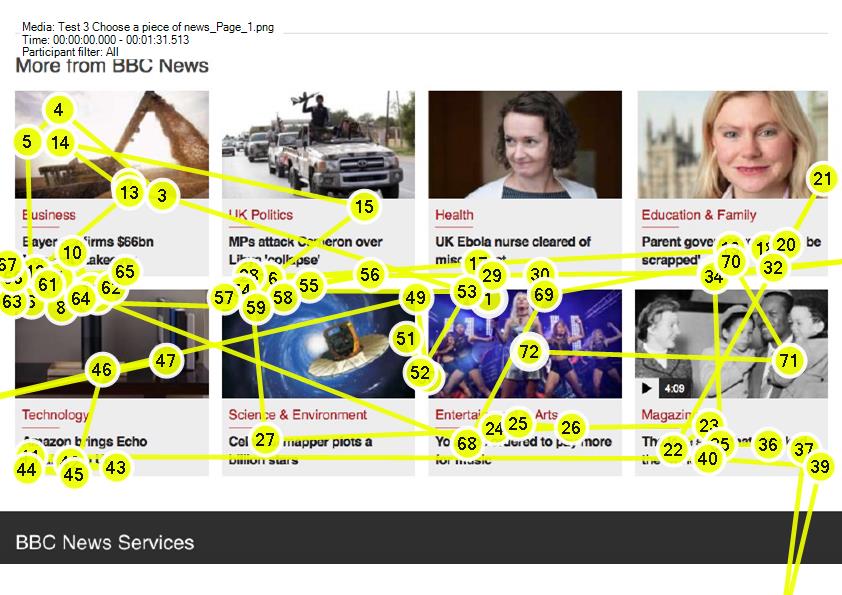

Supplement: Multimedia component 1 [file mmc1.zip › Data Data in Brief/4 Visual Data- Splited/Document 2/3 rec01.jpg]

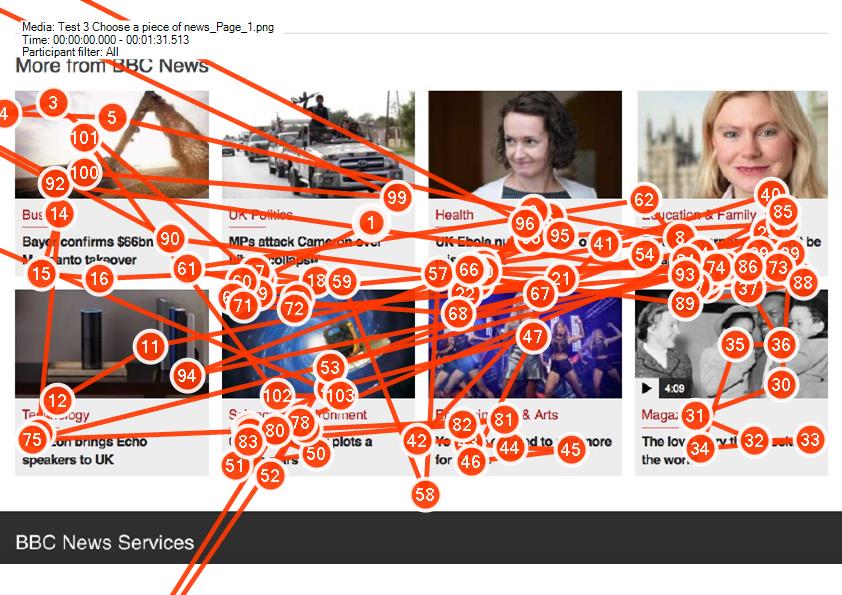

Supplement: Multimedia component 1 [file mmc1.zip › Data Data in Brief/4 Visual Data- Splited/Document 2/3 rec02.jpg]

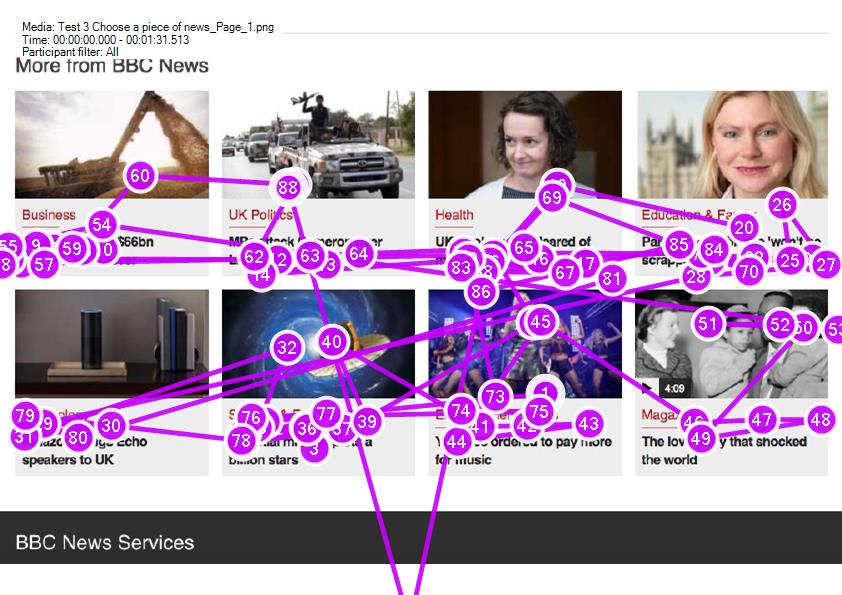

Supplement: Multimedia component 1 [file mmc1.zip › Data Data in Brief/4 Visual Data- Splited/Document 2/3 rec03.jpg]

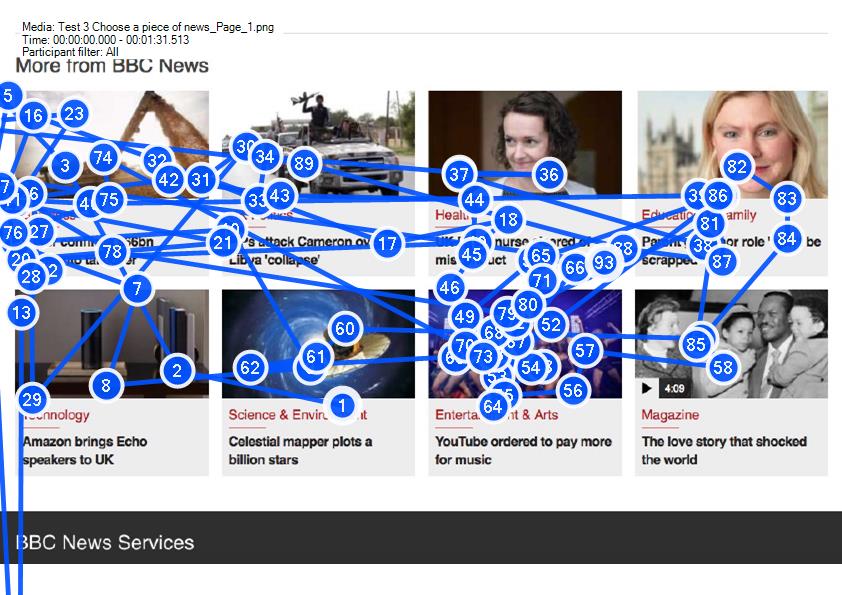

Supplement: Multimedia component 1 [file mmc1.zip › Data Data in Brief/4 Visual Data- Splited/Document 2/3 rec04.jpg]

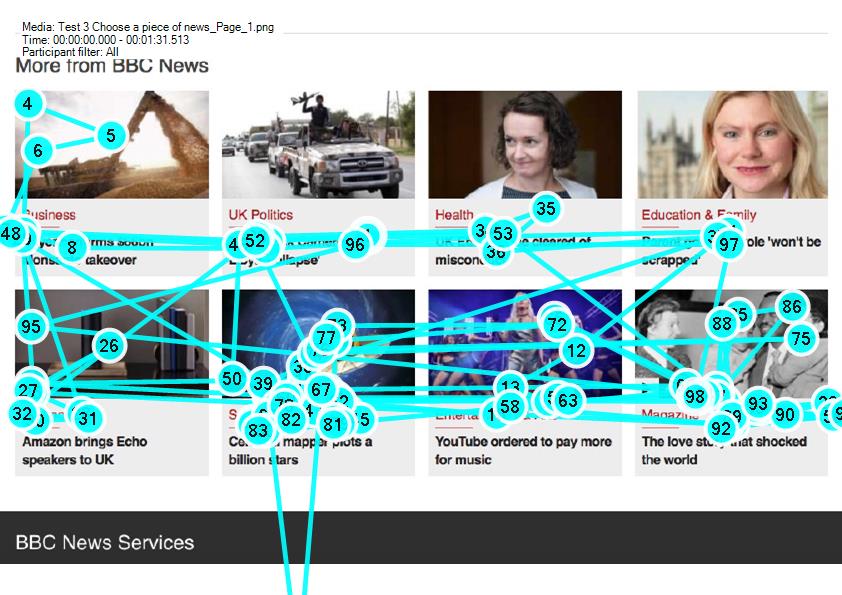

Supplement: Multimedia component 1 [file mmc1.zip › Data Data in Brief/4 Visual Data- Splited/Document 2/3 rec05.jpg]

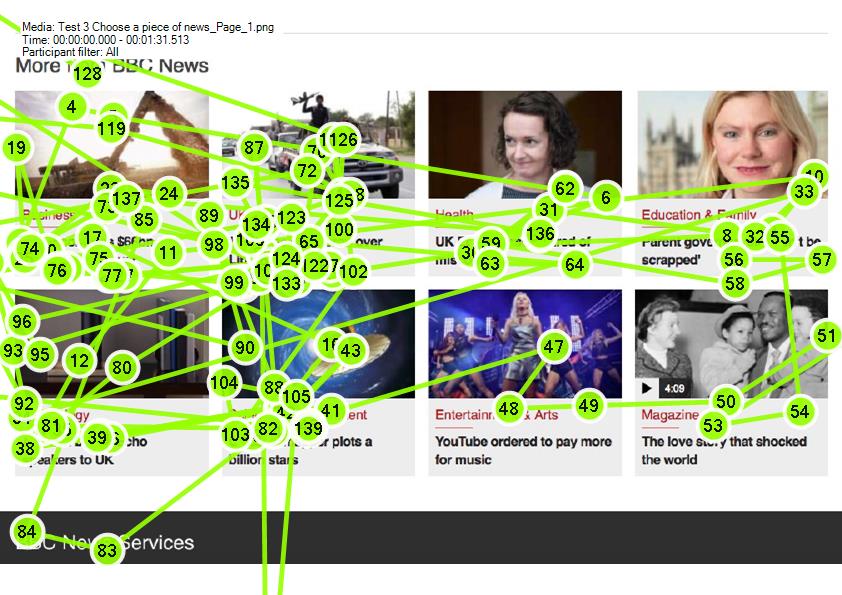

Supplement: Multimedia component 1 [file mmc1.zip › Data Data in Brief/4 Visual Data- Splited/Document 2/3 rec06.jpg]

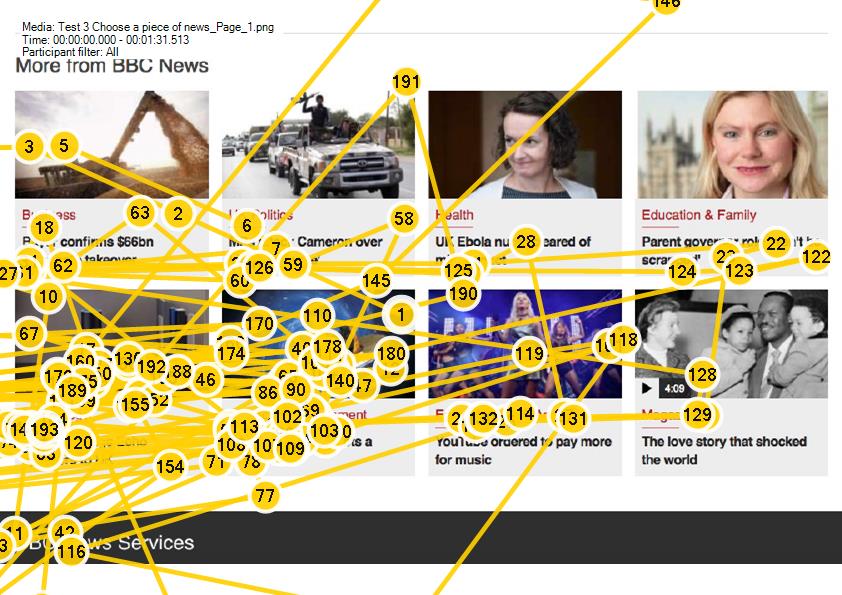

Supplement: Multimedia component 1 [file mmc1.zip › Data Data in Brief/4 Visual Data- Splited/Document 2/3 rec07.jpg]

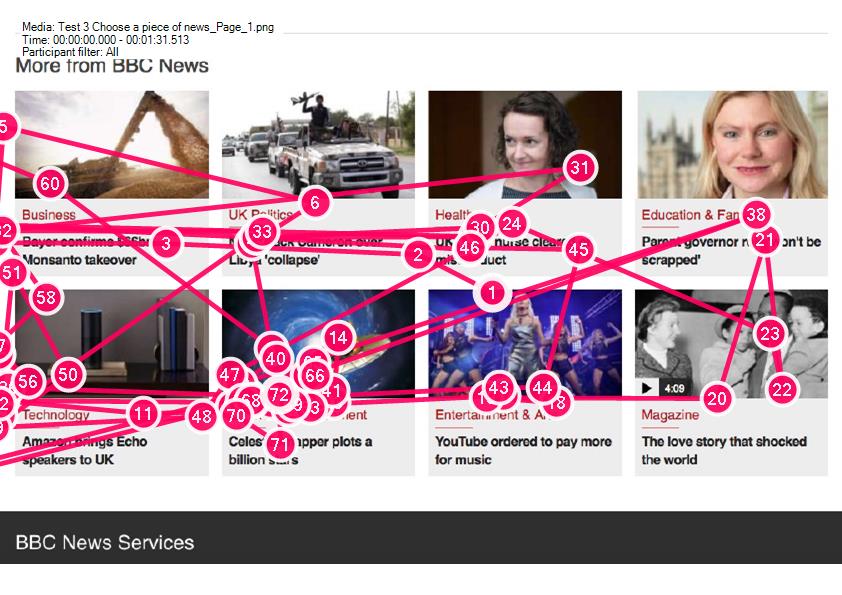

Supplement: Multimedia component 1 [file mmc1.zip › Data Data in Brief/4 Visual Data- Splited/Document 2/3 rec08.jpg]

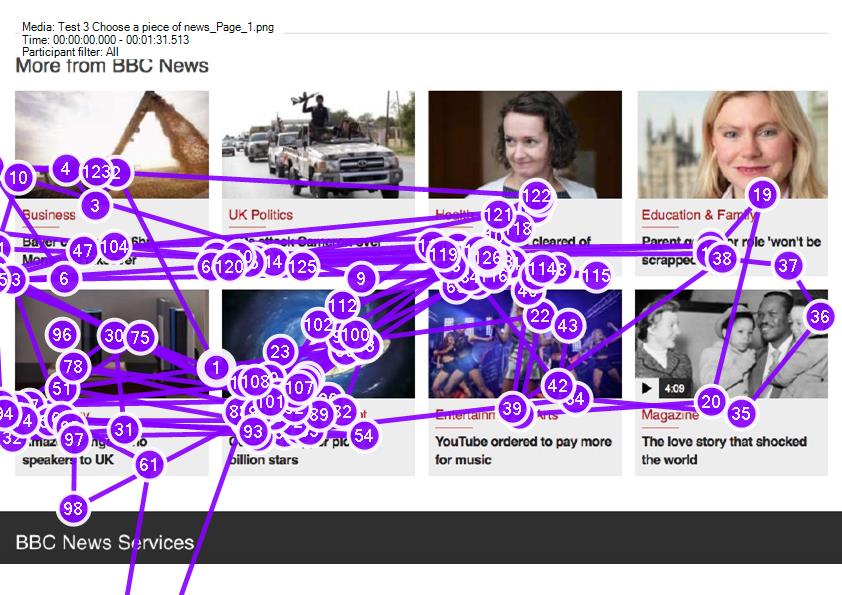

Supplement: Multimedia component 1 [file mmc1.zip › Data Data in Brief/4 Visual Data- Splited/Document 2/3 rec09.jpg]

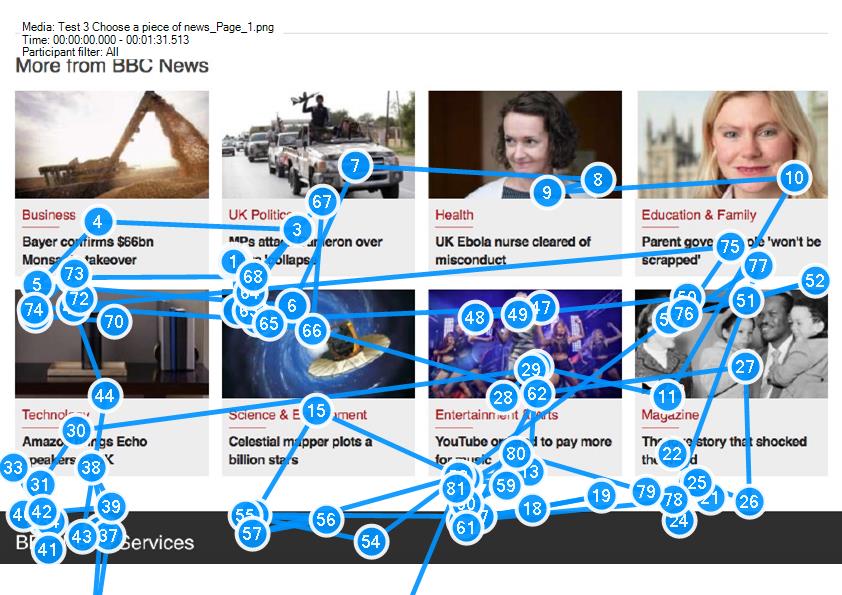

Supplement: Multimedia component 1 [file mmc1.zip › Data Data in Brief/4 Visual Data- Splited/Document 2/3 rec10.jpg]

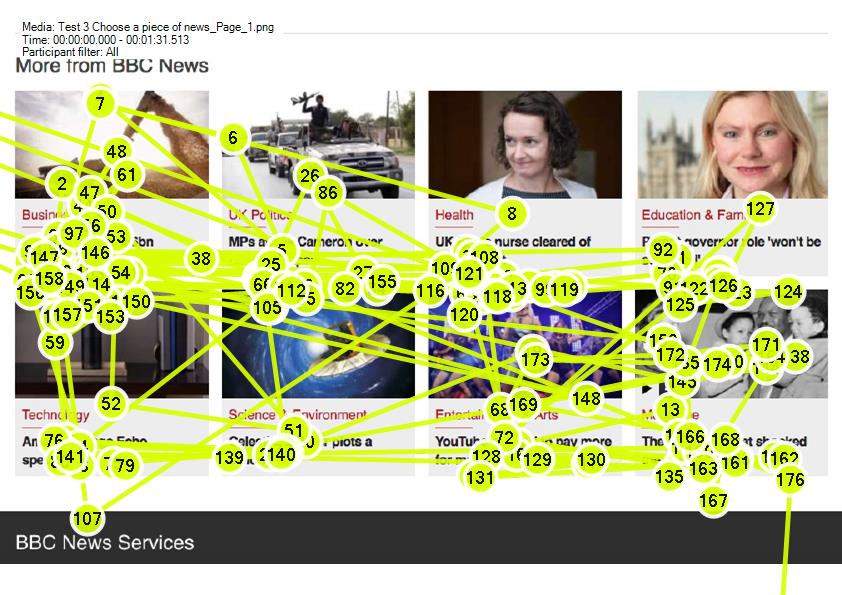

Supplement: Multimedia component 1 [file mmc1.zip › Data Data in Brief/4 Visual Data- Splited/Document 2/3 rec11.jpg]

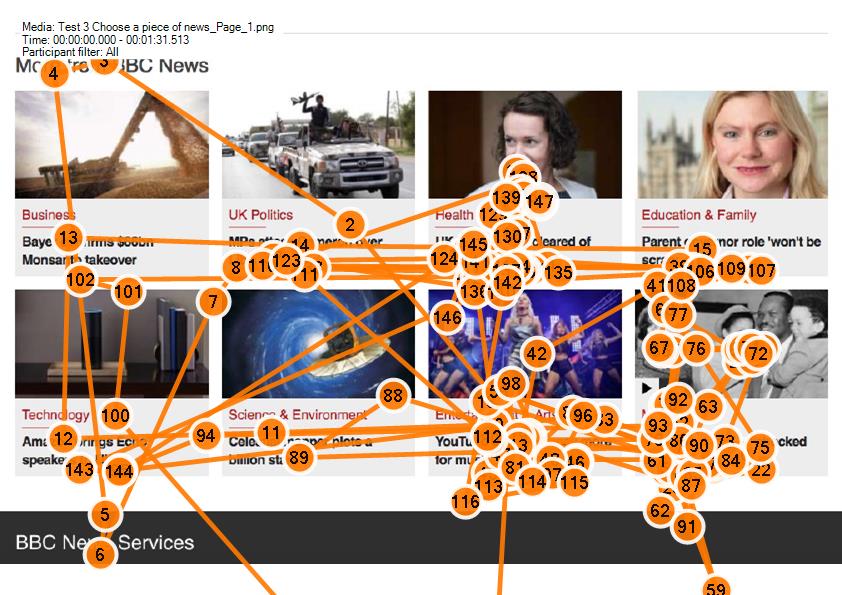

Supplement: Multimedia component 1 [file mmc1.zip › Data Data in Brief/4 Visual Data- Splited/Document 2/3 rec12.jpg]

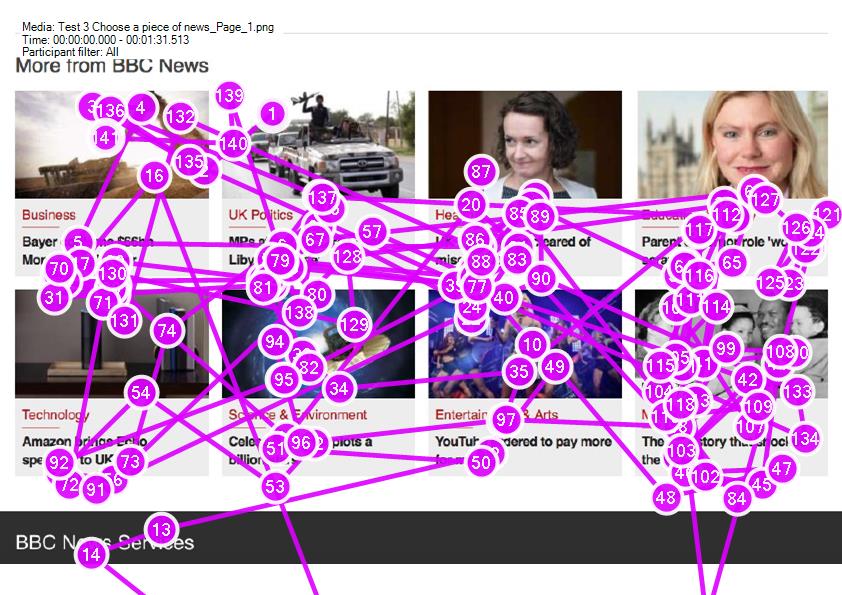

Supplement: Multimedia component 1 [file mmc1.zip › Data Data in Brief/4 Visual Data- Splited/Document 2/3 rec13.jpg]

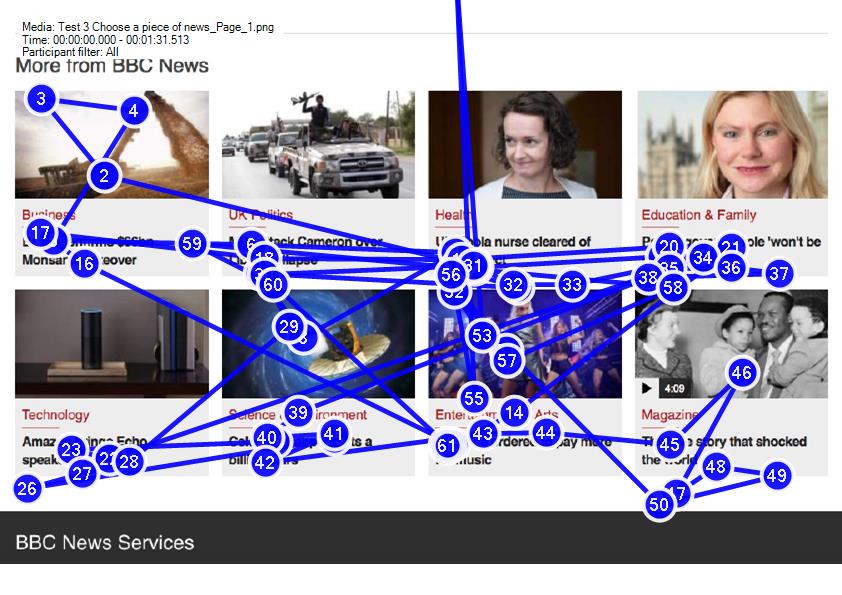

Supplement: Multimedia component 1 [file mmc1.zip › Data Data in Brief/4 Visual Data- Splited/Document 2/3 rec14.jpg]

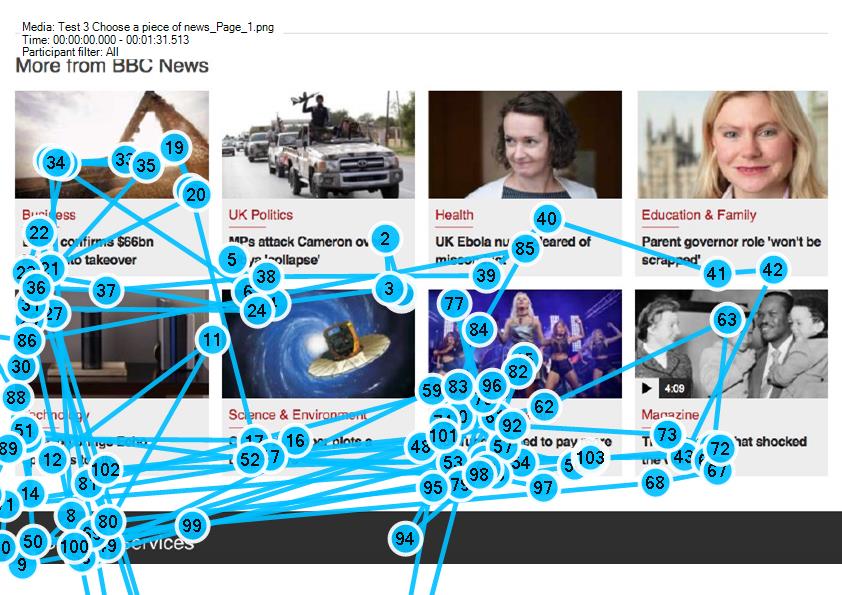

Supplement: Multimedia component 1 [file mmc1.zip › Data Data in Brief/4 Visual Data- Splited/Document 2/3 rec15.jpg]

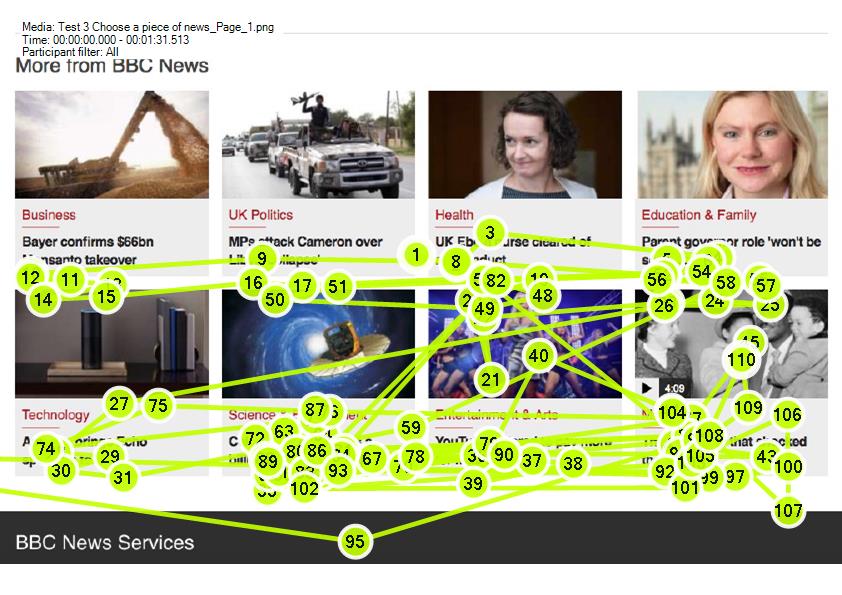

Supplement: Multimedia component 1 [file mmc1.zip › Data Data in Brief/4 Visual Data- Splited/Document 2/3 rec16.jpg]

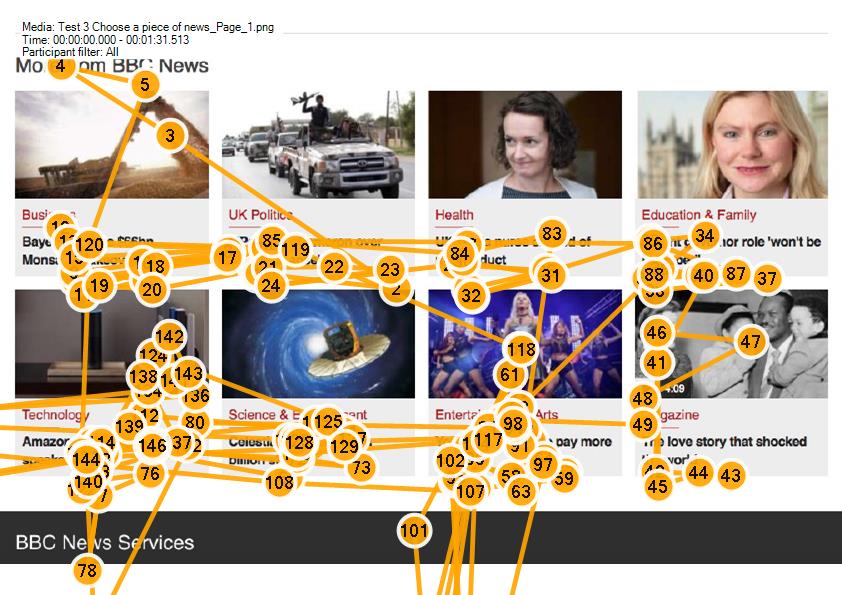

Supplement: Multimedia component 1 [file mmc1.zip › Data Data in Brief/4 Visual Data- Splited/Document 2/3 rec17.jpg]

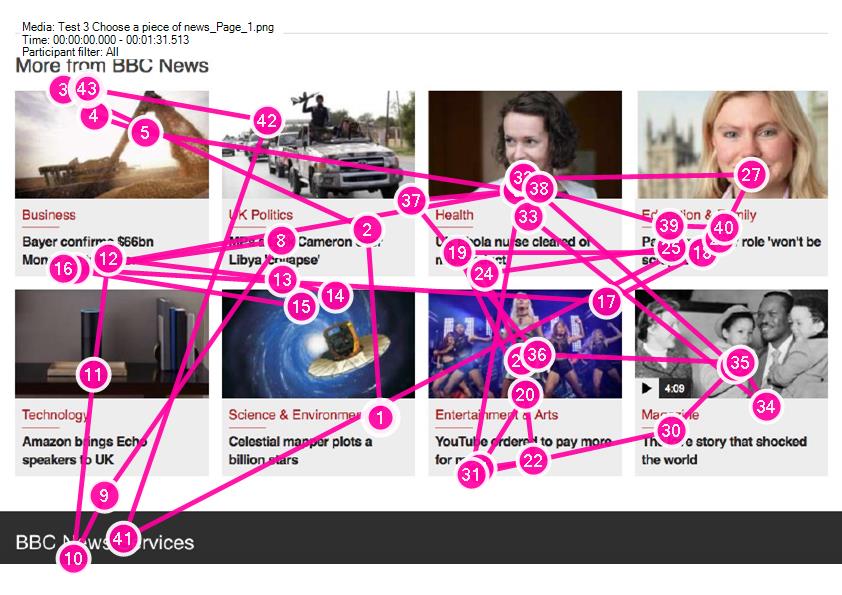

Supplement: Multimedia component 1 [file mmc1.zip › Data Data in Brief/4 Visual Data- Splited/Document 2/3 rec18.jpg]

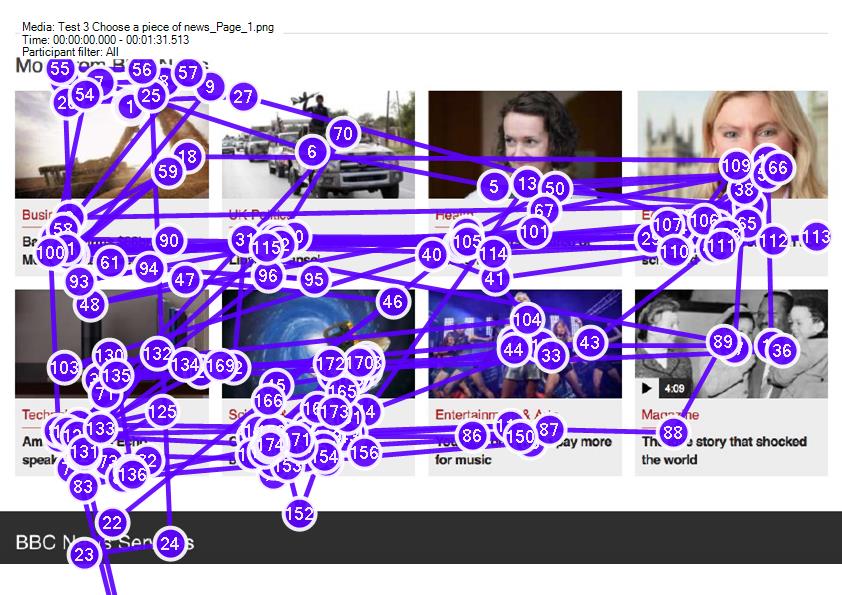

Supplement: Multimedia component 1 [file mmc1.zip › Data Data in Brief/4 Visual Data- Splited/Document 2/3 rec19.jpg]

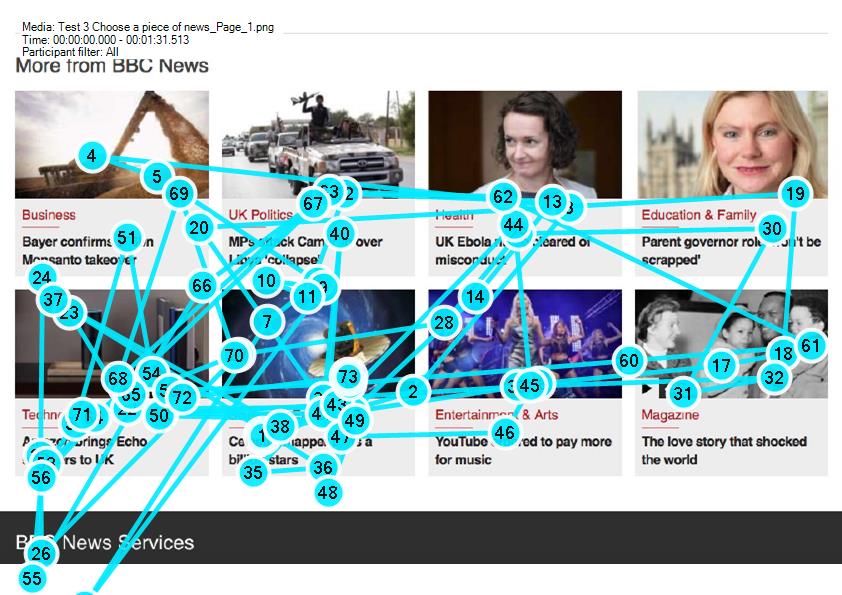

Supplement: Multimedia component 1 [file mmc1.zip › Data Data in Brief/4 Visual Data- Splited/Document 2/3 rec20.jpg]

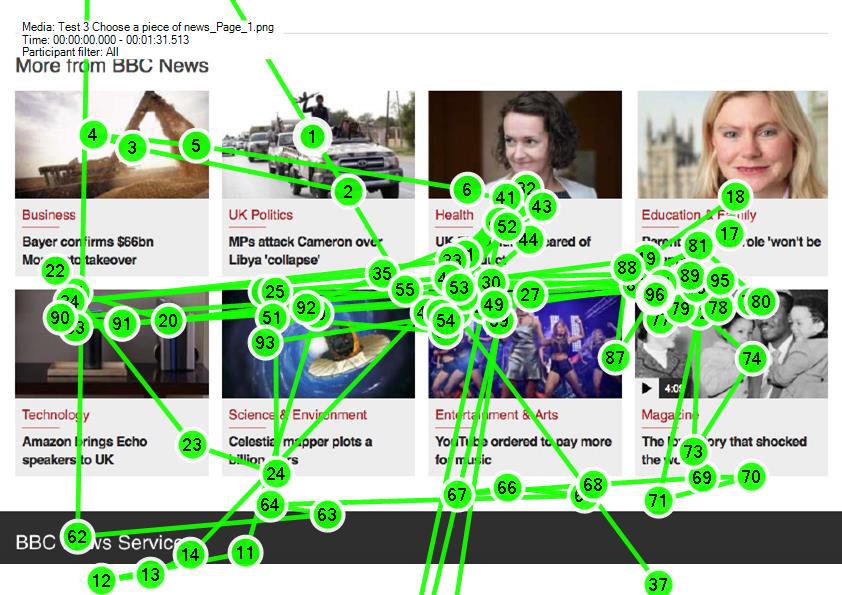

Supplement: Multimedia component 1 [file mmc1.zip › Data Data in Brief/4 Visual Data- Splited/Document 2/3 rec21.jpg]

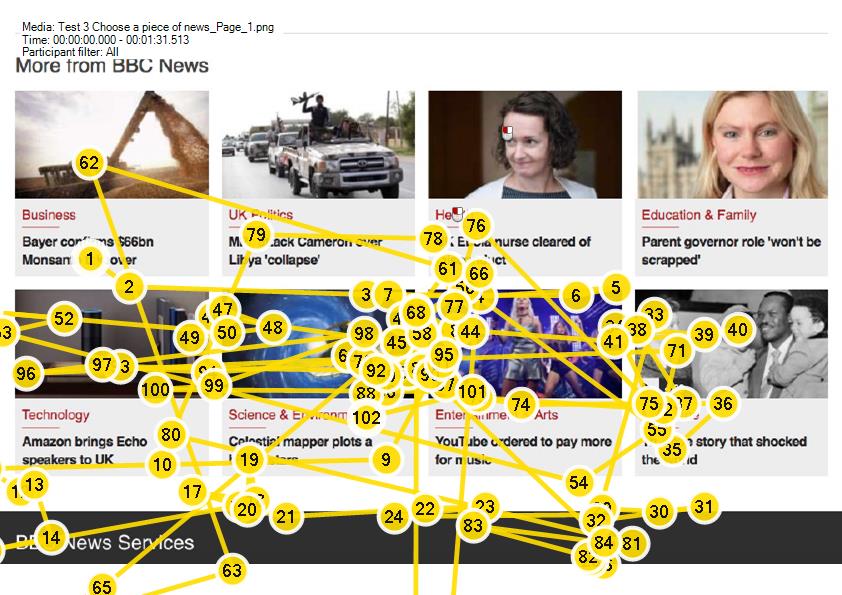

Supplement: Multimedia component 1 [file mmc1.zip › Data Data in Brief/4 Visual Data- Splited/Document 2/3 rec22.jpg]

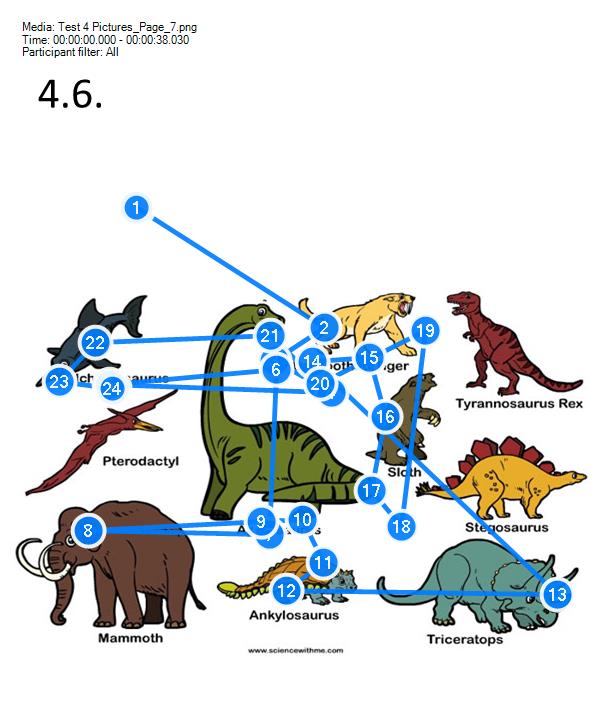

Supplement: Multimedia component 1 [file mmc1.zip › Data Data in Brief/4 Visual Data- Splited/Document 3/4-2 rec pilot.jpg]

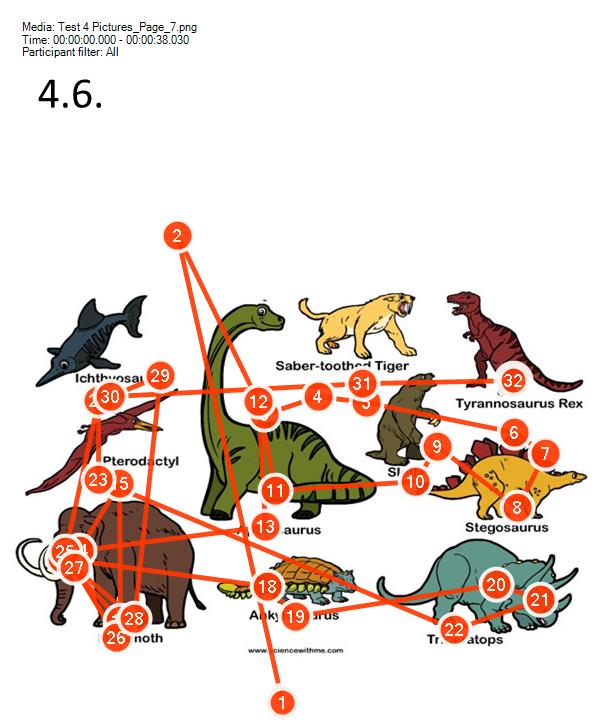

Supplement: Multimedia component 1 [file mmc1.zip › Data Data in Brief/4 Visual Data- Splited/Document 3/4-2 rec02.jpg]

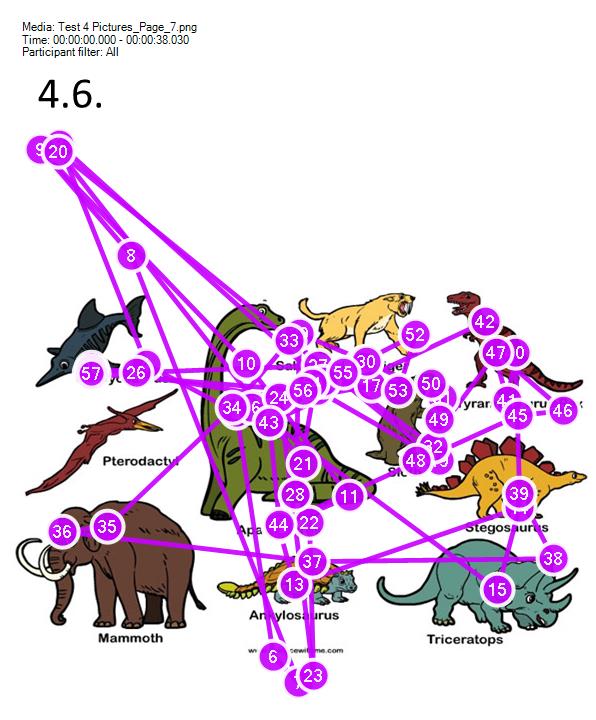

Supplement: Multimedia component 1 [file mmc1.zip › Data Data in Brief/4 Visual Data- Splited/Document 3/4-2 rec03.jpg]

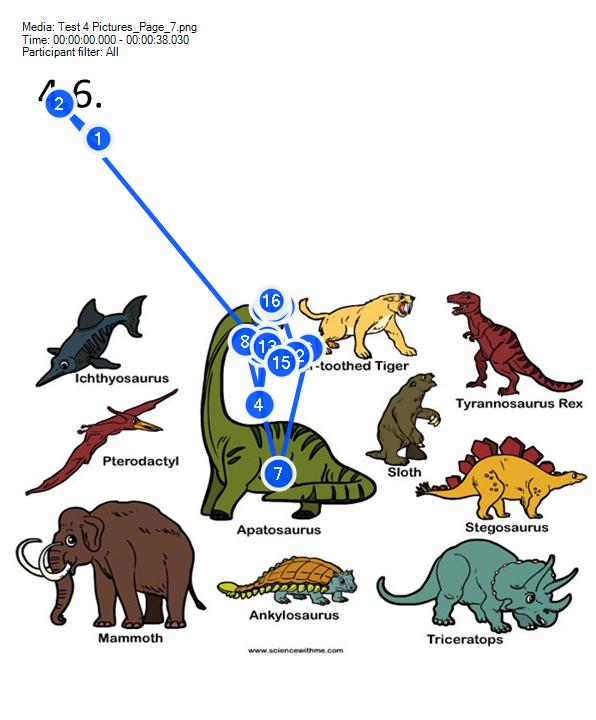

Supplement: Multimedia component 1 [file mmc1.zip › Data Data in Brief/4 Visual Data- Splited/Document 3/4-2 rec04.jpg]

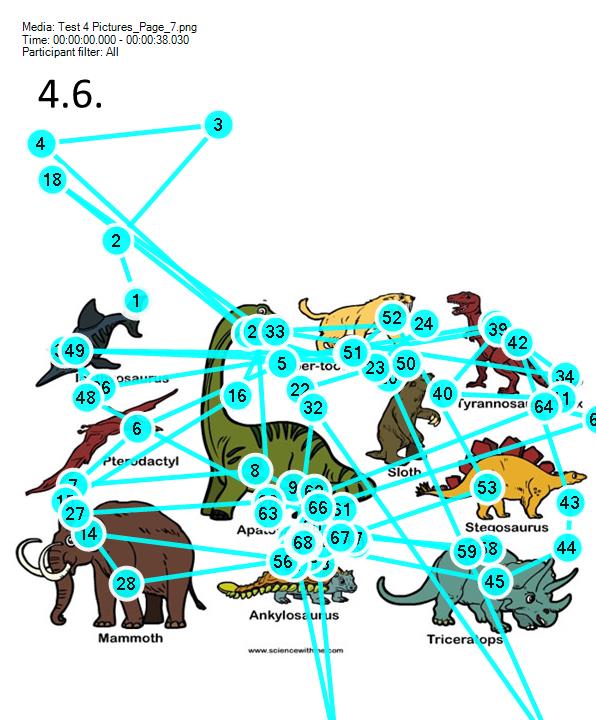

Supplement: Multimedia component 1 [file mmc1.zip › Data Data in Brief/4 Visual Data- Splited/Document 3/4-2 rec05.jpg]

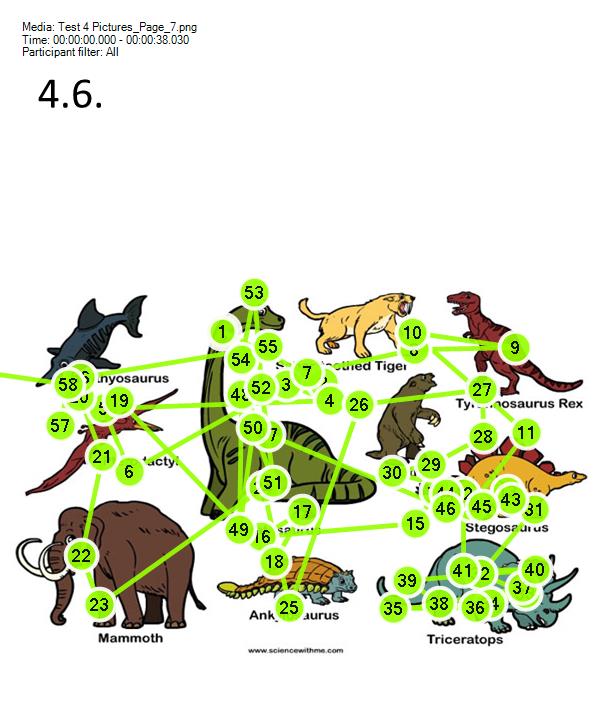

Supplement: Multimedia component 1 [file mmc1.zip › Data Data in Brief/4 Visual Data- Splited/Document 3/4-2 rec06.jpg]

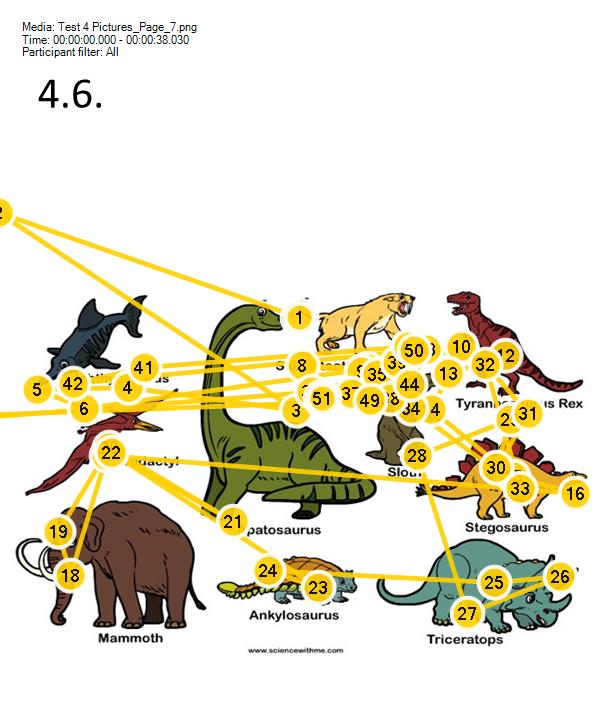

Supplement: Multimedia component 1 [file mmc1.zip › Data Data in Brief/4 Visual Data- Splited/Document 3/4-2 rec07.jpg]

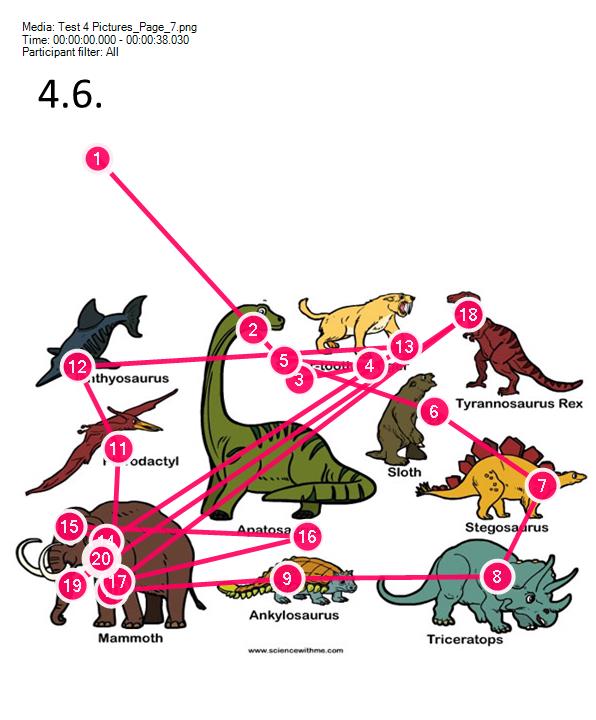

Supplement: Multimedia component 1 [file mmc1.zip › Data Data in Brief/4 Visual Data- Splited/Document 3/4-2 rec08.jpg]

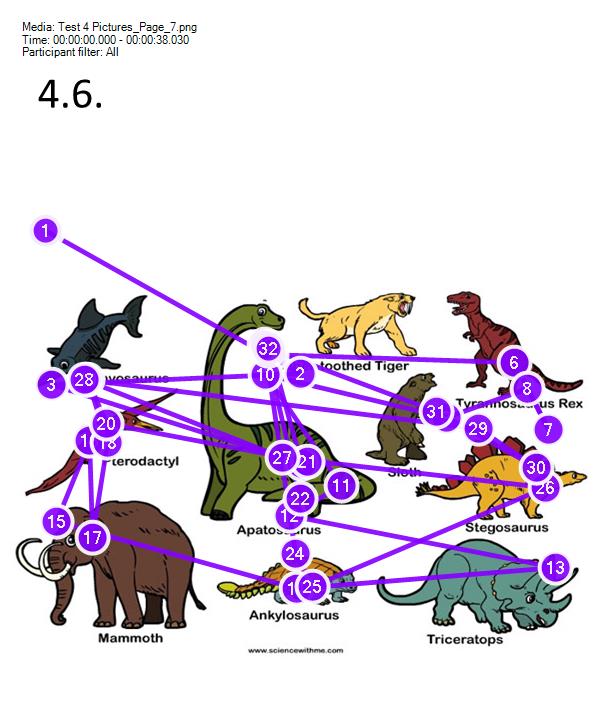

Supplement: Multimedia component 1 [file mmc1.zip › Data Data in Brief/4 Visual Data- Splited/Document 3/4-2 rec09.jpg]

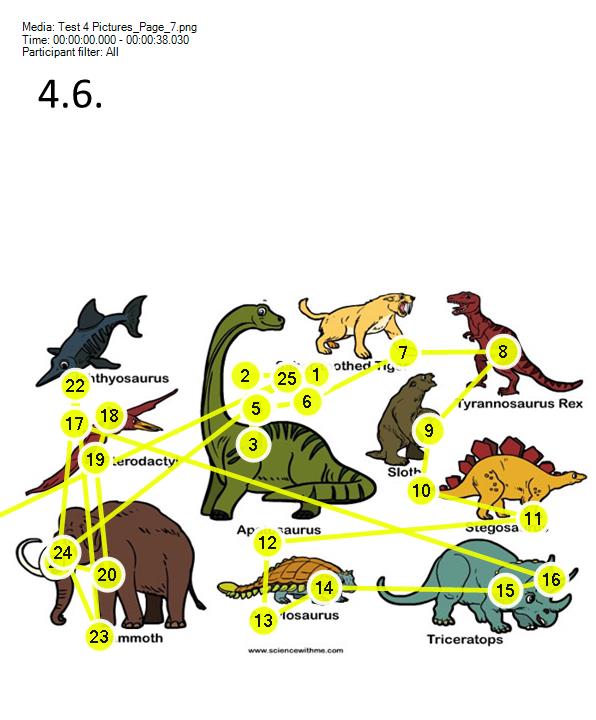

Supplement: Multimedia component 1 [file mmc1.zip › Data Data in Brief/4 Visual Data- Splited/Document 3/4-2 rec1.jpg]

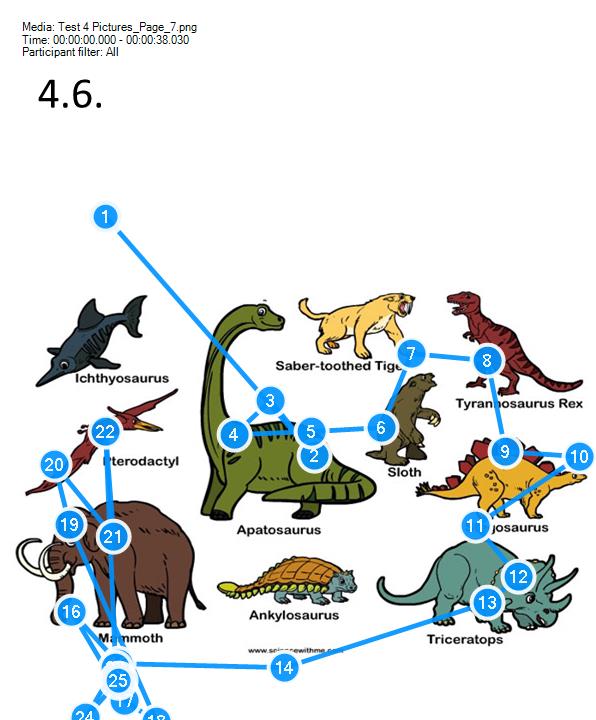

Supplement: Multimedia component 1 [file mmc1.zip › Data Data in Brief/4 Visual Data- Splited/Document 3/4-2 rec10.jpg]

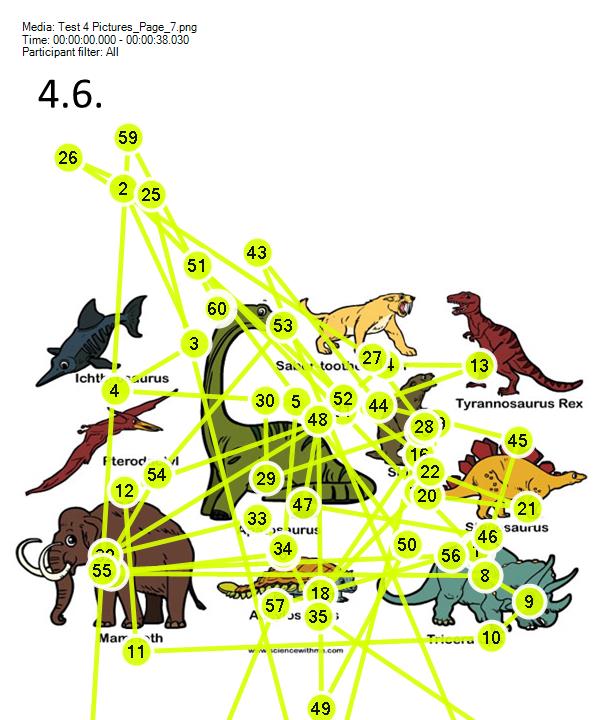

Supplement: Multimedia component 1 [file mmc1.zip › Data Data in Brief/4 Visual Data- Splited/Document 3/4-2 rec11.jpg]

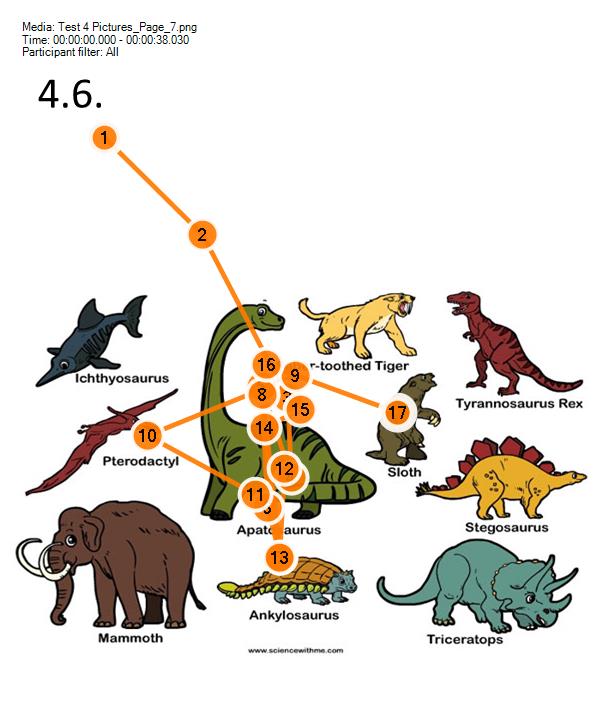

Supplement: Multimedia component 1 [file mmc1.zip › Data Data in Brief/4 Visual Data- Splited/Document 3/4-2 rec12.jpg]

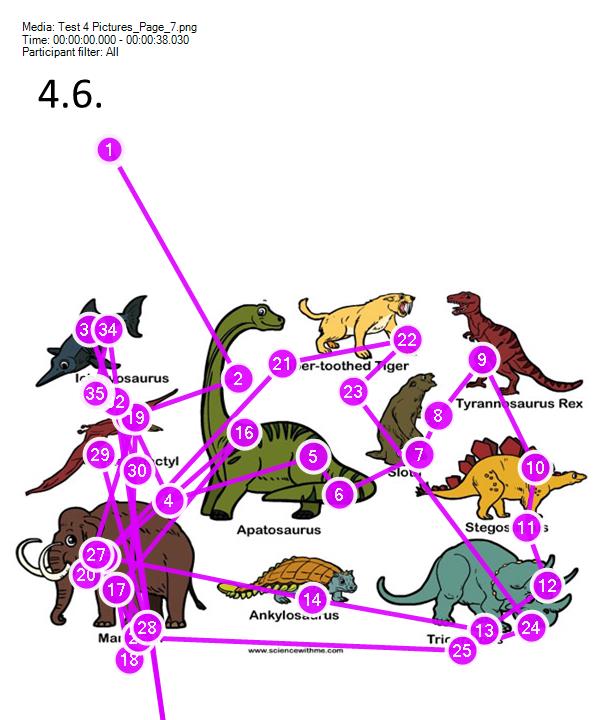

Supplement: Multimedia component 1 [file mmc1.zip › Data Data in Brief/4 Visual Data- Splited/Document 3/4-2 rec13.jpg]

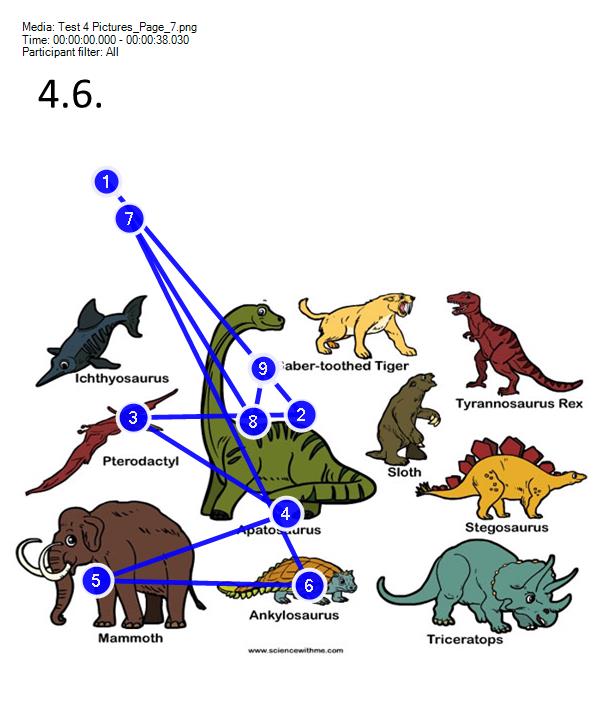

Supplement: Multimedia component 1 [file mmc1.zip › Data Data in Brief/4 Visual Data- Splited/Document 3/4-2 rec14.jpg]

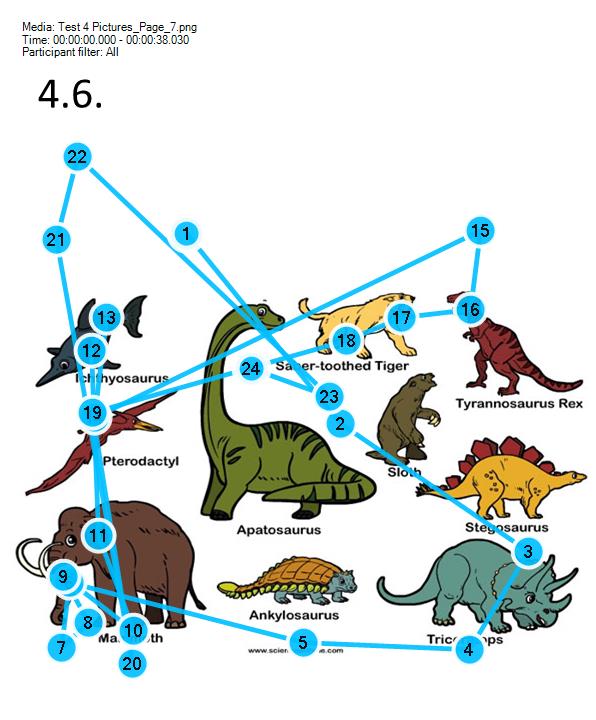

Supplement: Multimedia component 1 [file mmc1.zip › Data Data in Brief/4 Visual Data- Splited/Document 3/4-2 rec15.jpg]

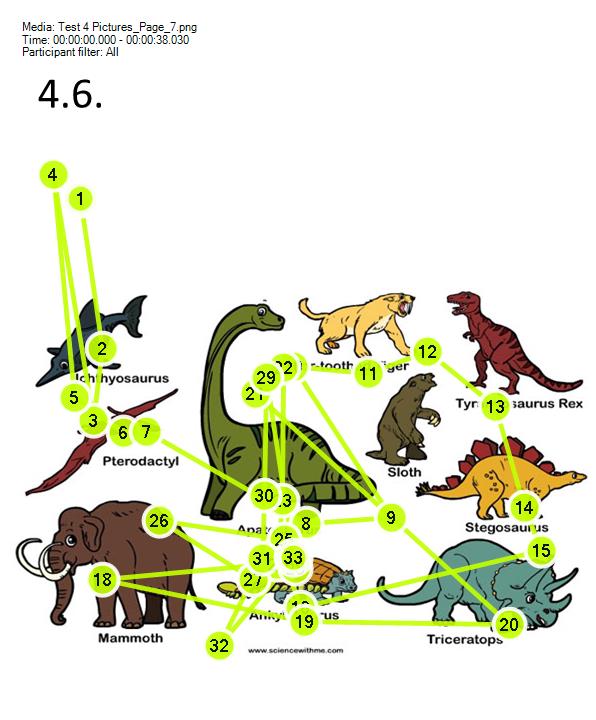

Supplement: Multimedia component 1 [file mmc1.zip › Data Data in Brief/4 Visual Data- Splited/Document 3/4-2 rec16.jpg]

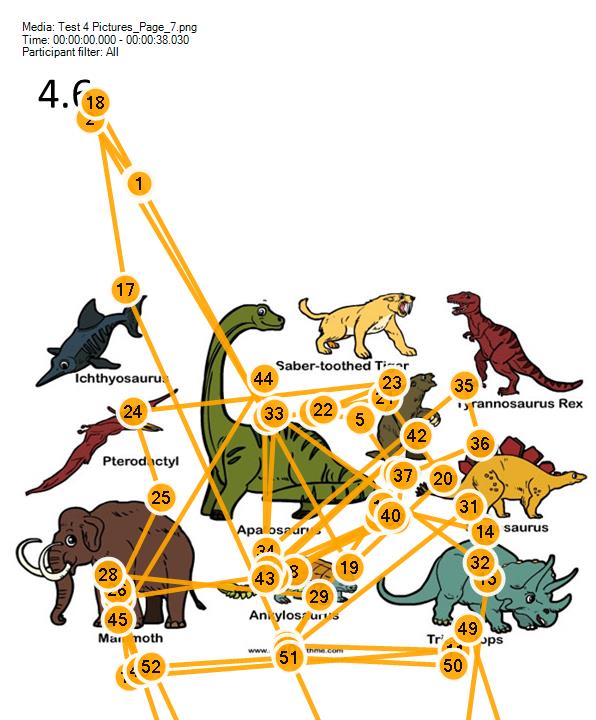

Supplement: Multimedia component 1 [file mmc1.zip › Data Data in Brief/4 Visual Data- Splited/Document 3/4-2 rec17.jpg]

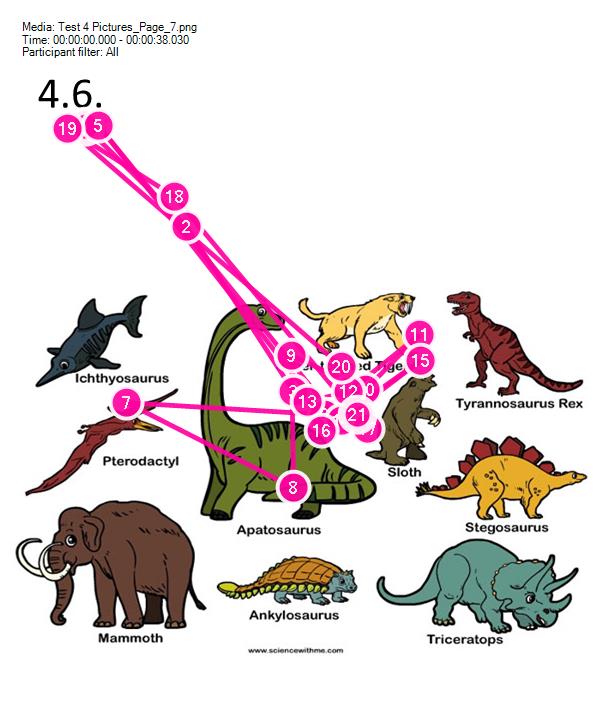

Supplement: Multimedia component 1 [file mmc1.zip › Data Data in Brief/4 Visual Data- Splited/Document 3/4-2 rec18.jpg]

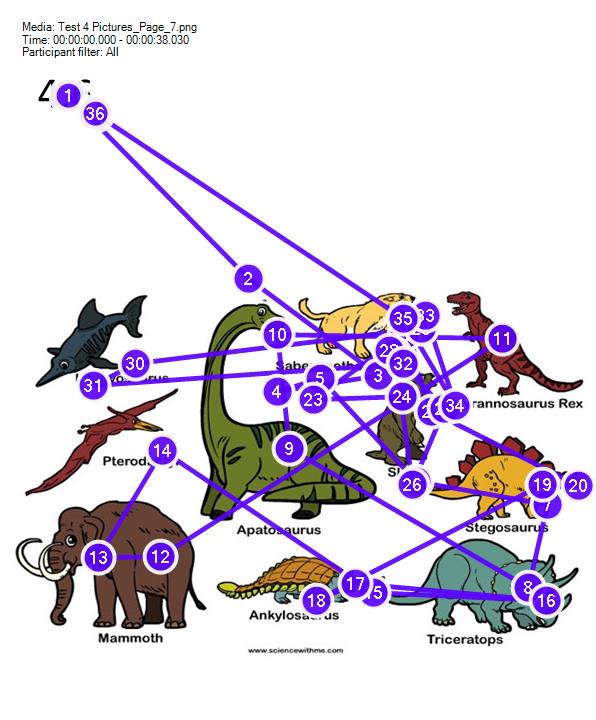

Supplement: Multimedia component 1 [file mmc1.zip › Data Data in Brief/4 Visual Data- Splited/Document 3/4-2 rec19.jpg]

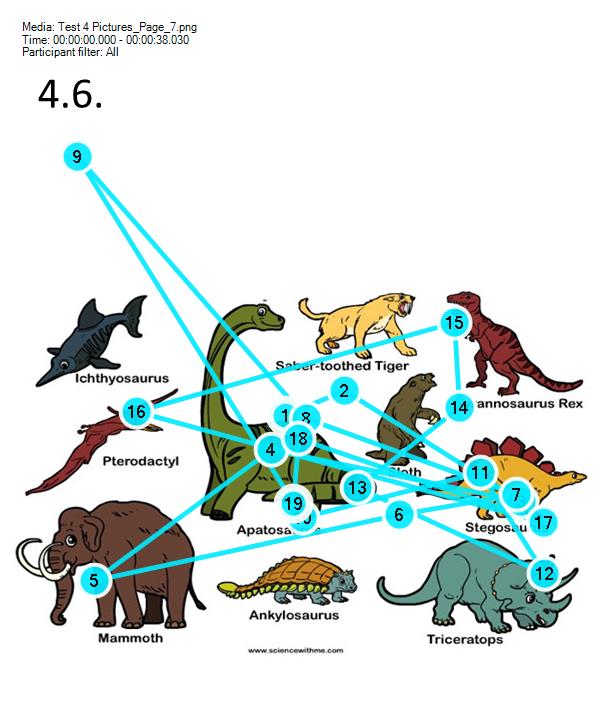

Supplement: Multimedia component 1 [file mmc1.zip › Data Data in Brief/4 Visual Data- Splited/Document 3/4-2 rec20.jpg]

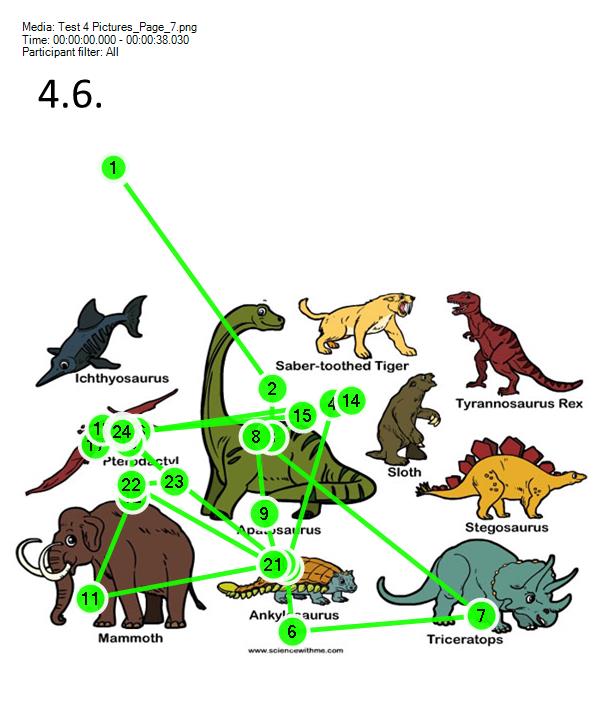

Supplement: Multimedia component 1 [file mmc1.zip › Data Data in Brief/4 Visual Data- Splited/Document 3/4-2 rec21.jpg]

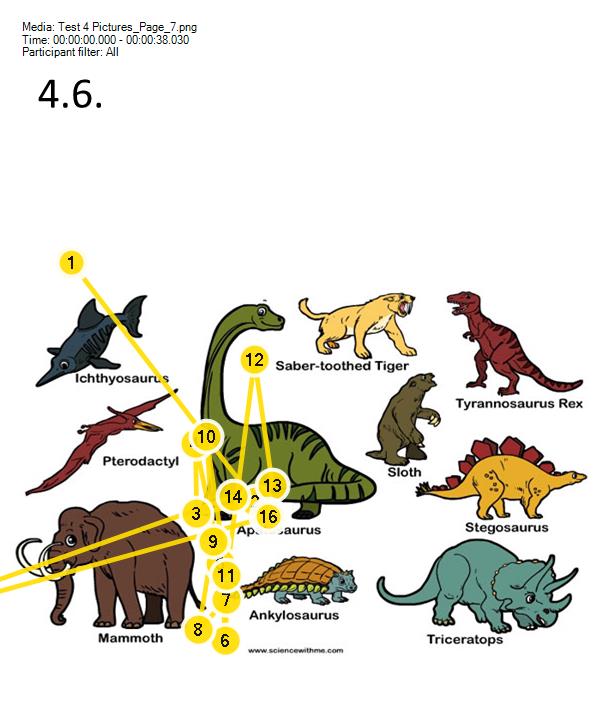

Supplement: Multimedia component 1 [file mmc1.zip › Data Data in Brief/4 Visual Data- Splited/Document 3/4-2 rec22.jpg]

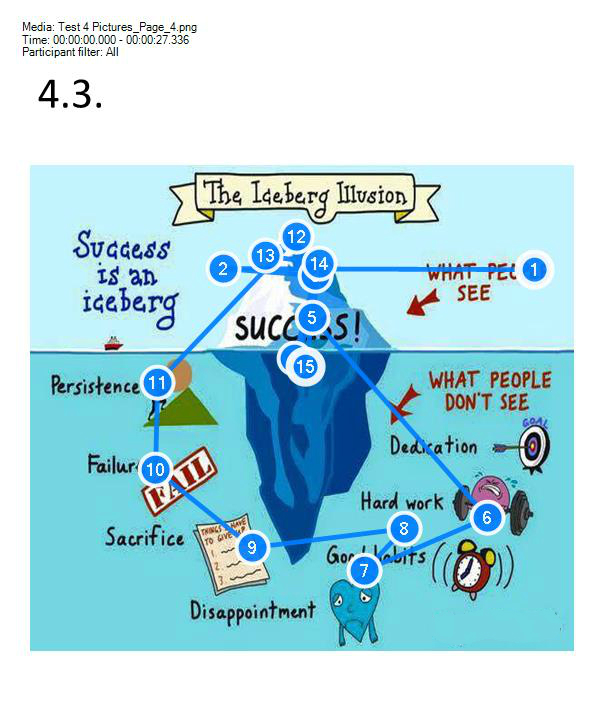

Supplement: Multimedia component 1 [file mmc1.zip › Data Data in Brief/4 Visual Data- Splited/Document 4/4-1 rec pilot.jpg]

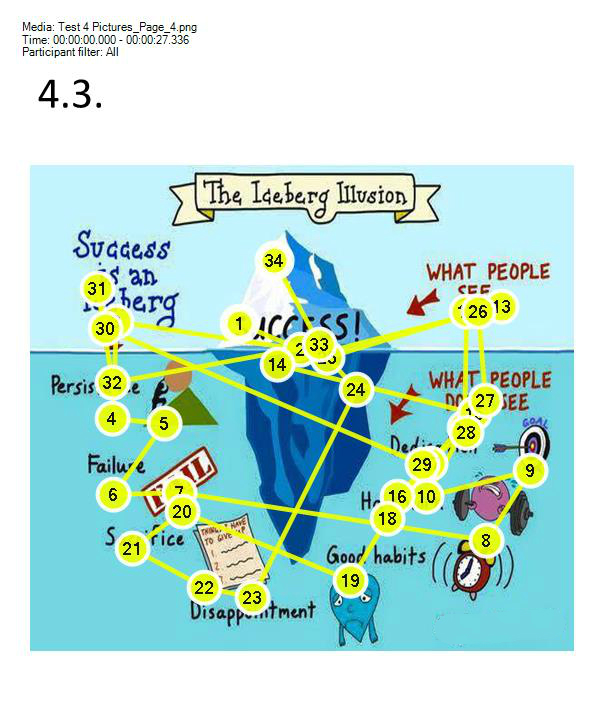

Supplement: Multimedia component 1 [file mmc1.zip › Data Data in Brief/4 Visual Data- Splited/Document 4/4-1 rec01.jpg]

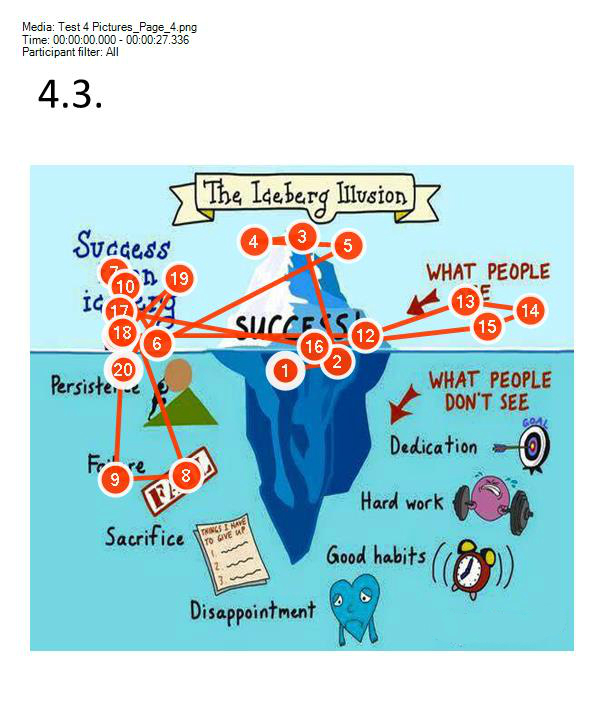

Supplement: Multimedia component 1 [file mmc1.zip › Data Data in Brief/4 Visual Data- Splited/Document 4/4-1 rec02.jpg]

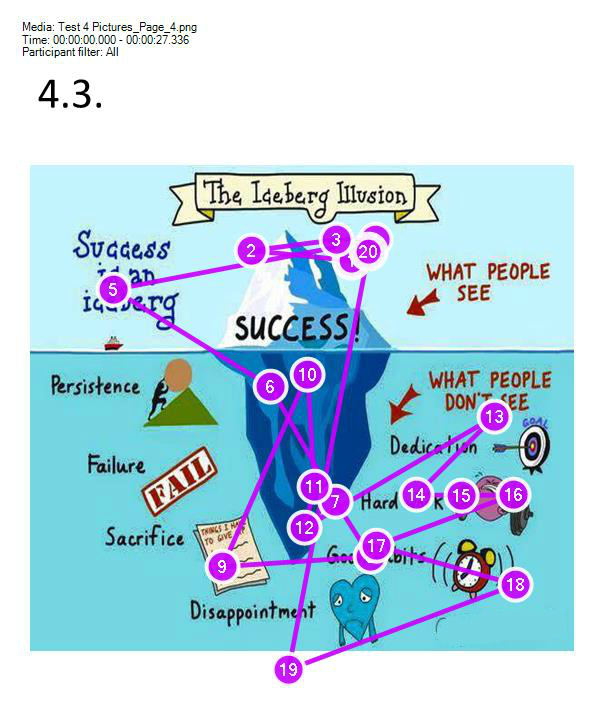

Supplement: Multimedia component 1 [file mmc1.zip › Data Data in Brief/4 Visual Data- Splited/Document 4/4-1 rec03.jpg]

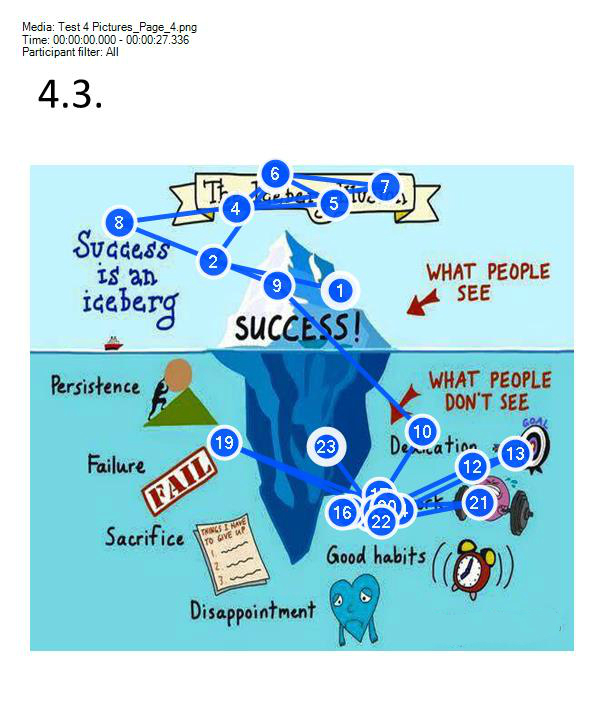

Supplement: Multimedia component 1 [file mmc1.zip › Data Data in Brief/4 Visual Data- Splited/Document 4/4-1 rec04.jpg]

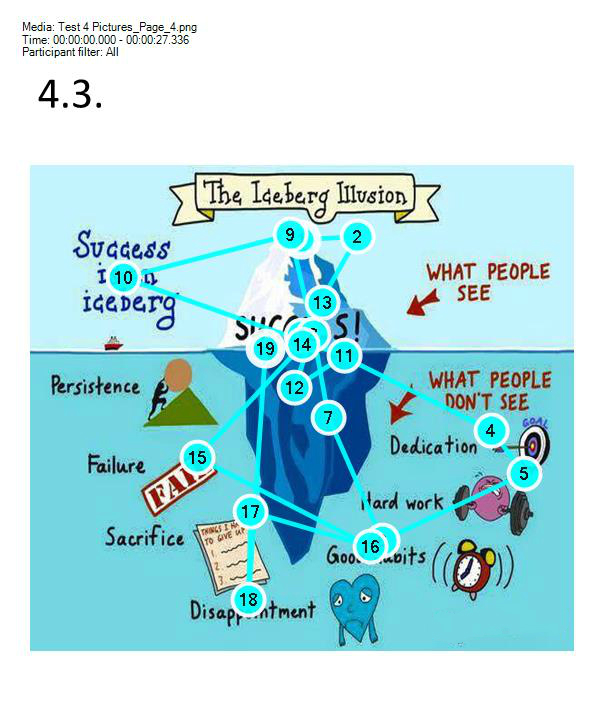

Supplement: Multimedia component 1 [file mmc1.zip › Data Data in Brief/4 Visual Data- Splited/Document 4/4-1 rec05.jpg]

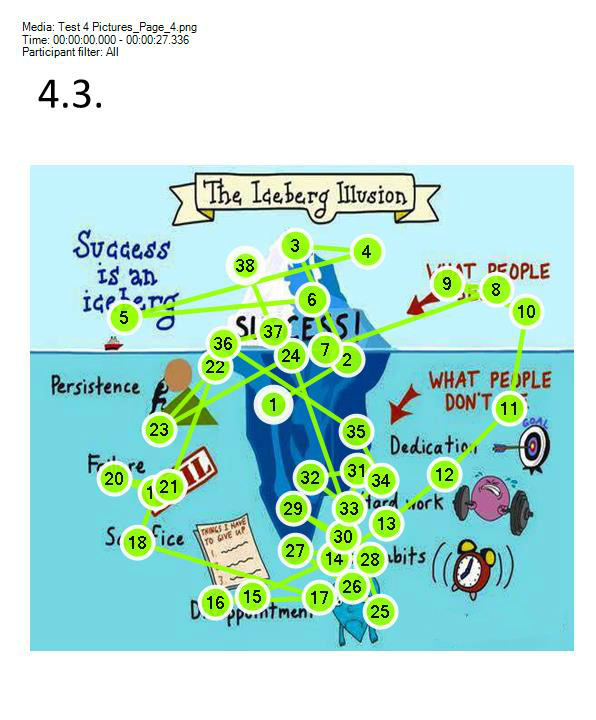

Supplement: Multimedia component 1 [file mmc1.zip › Data Data in Brief/4 Visual Data- Splited/Document 4/4-1 rec06.jpg]

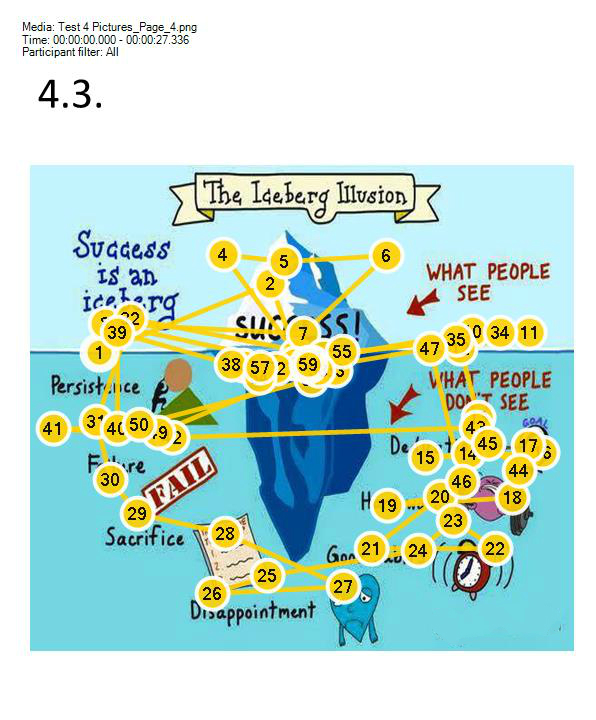

Supplement: Multimedia component 1 [file mmc1.zip › Data Data in Brief/4 Visual Data- Splited/Document 4/4-1 rec07.jpg]

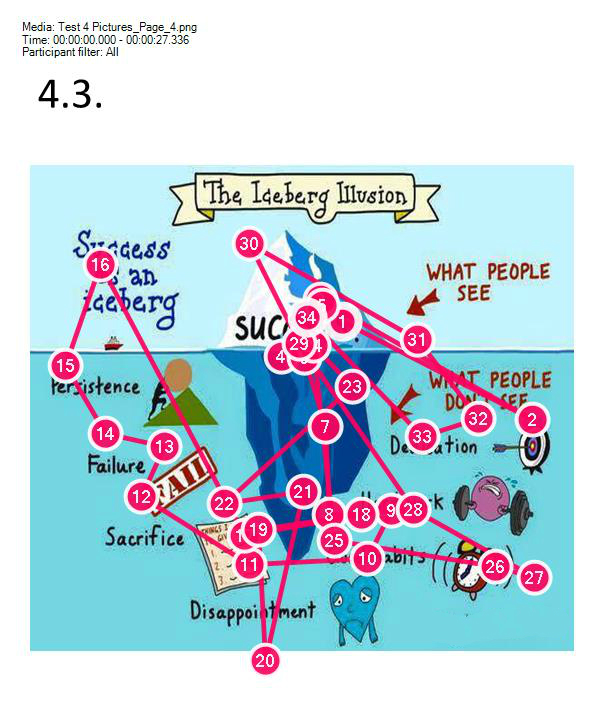

Supplement: Multimedia component 1 [file mmc1.zip › Data Data in Brief/4 Visual Data- Splited/Document 4/4-1 rec08.jpg]

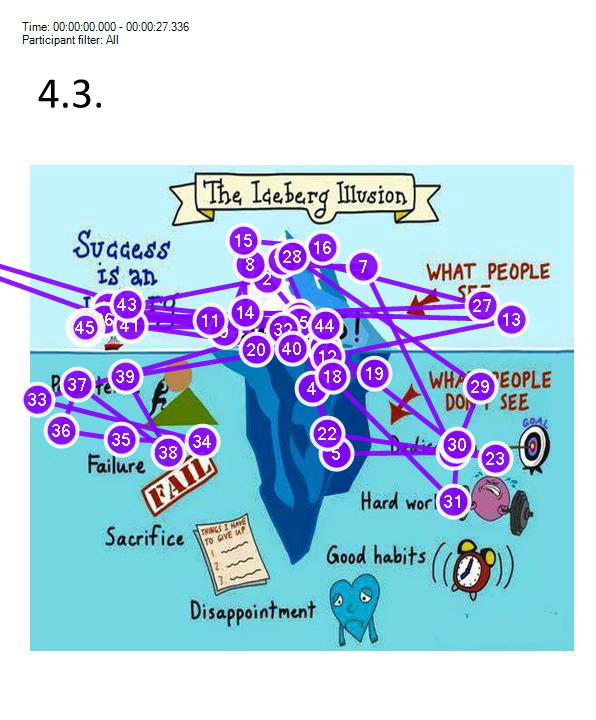

Supplement: Multimedia component 1 [file mmc1.zip › Data Data in Brief/4 Visual Data- Splited/Document 4/4-1 rec09.jpg]

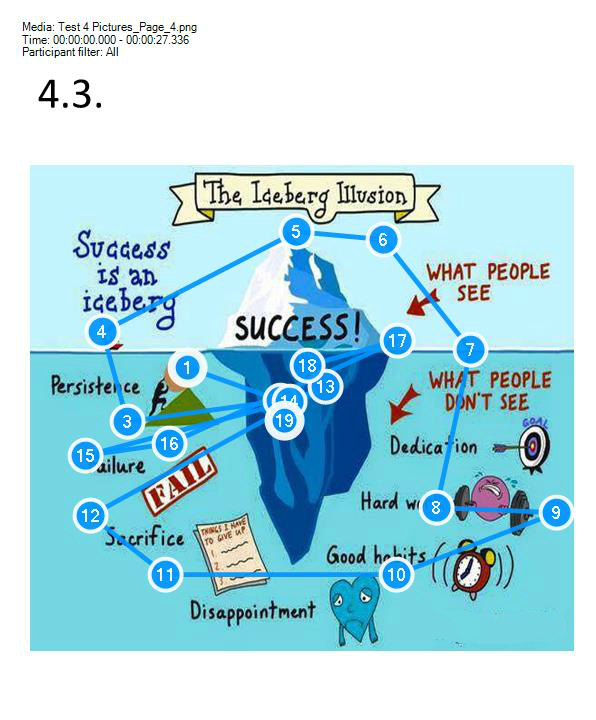

Supplement: Multimedia component 1 [file mmc1.zip › Data Data in Brief/4 Visual Data- Splited/Document 4/4-1 rec10.jpg]

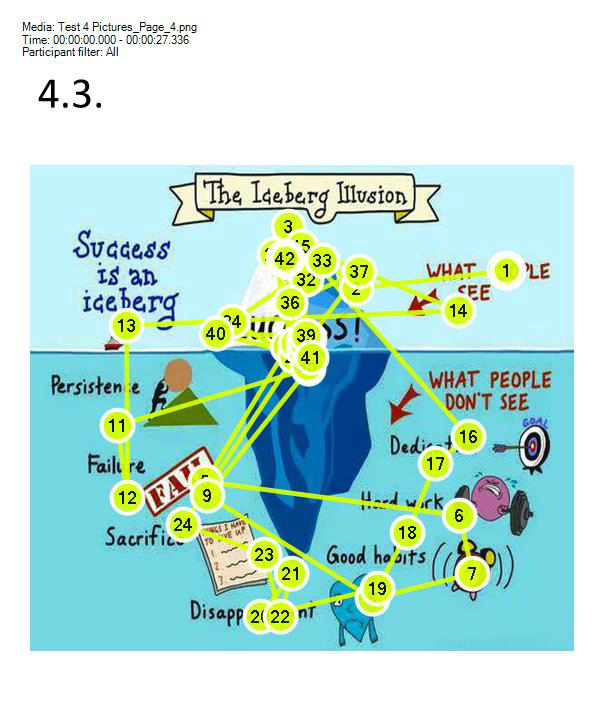

Supplement: Multimedia component 1 [file mmc1.zip › Data Data in Brief/4 Visual Data- Splited/Document 4/4-1 rec11.jpg]

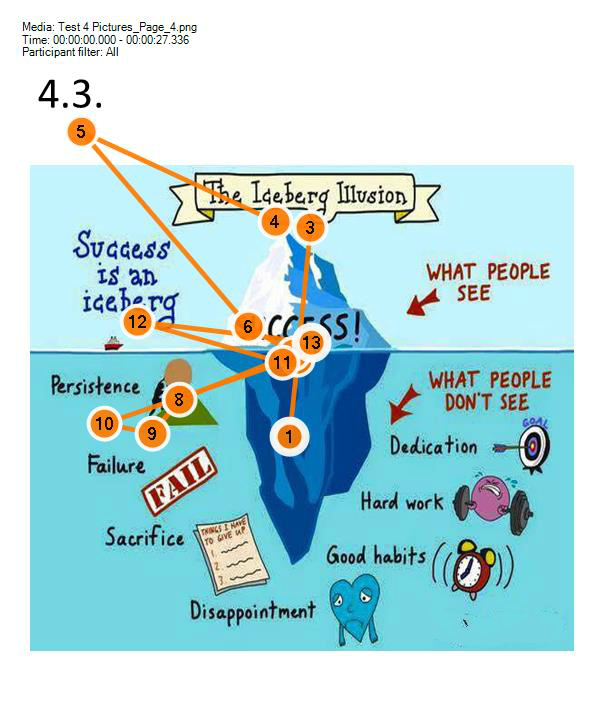

Supplement: Multimedia component 1 [file mmc1.zip › Data Data in Brief/4 Visual Data- Splited/Document 4/4-1 rec12.jpg]

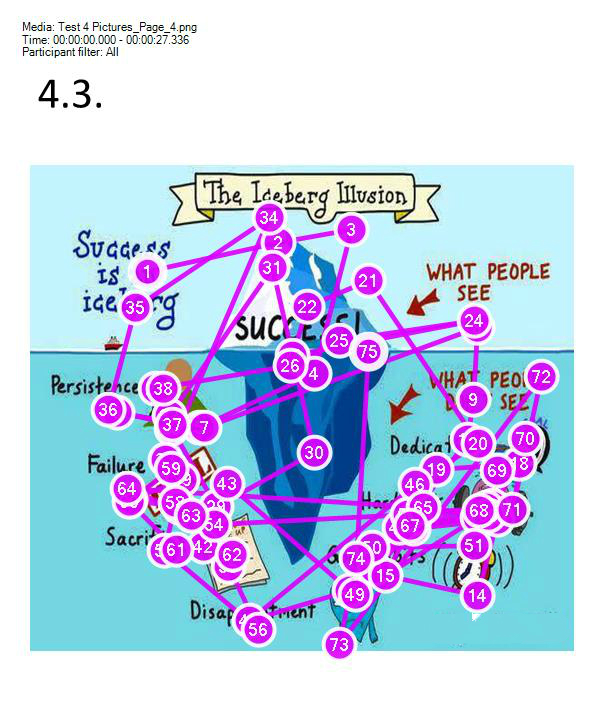

Supplement: Multimedia component 1 [file mmc1.zip › Data Data in Brief/4 Visual Data- Splited/Document 4/4-1 rec13.jpg]

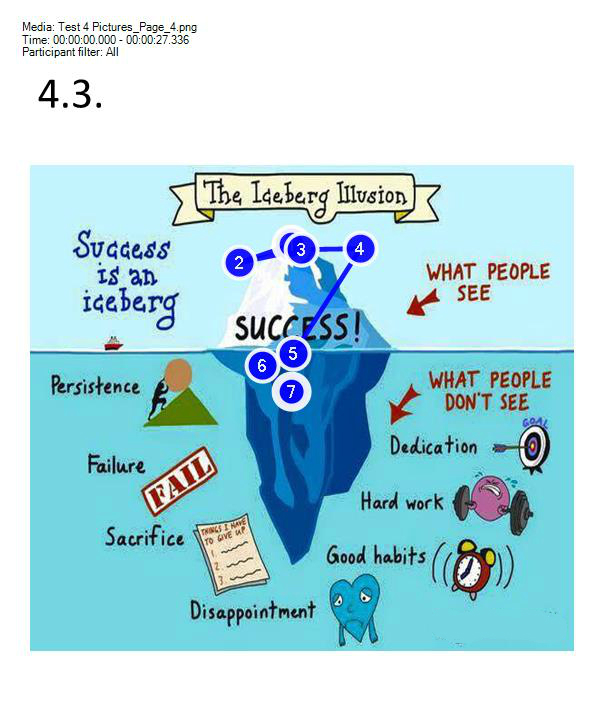

Supplement: Multimedia component 1 [file mmc1.zip › Data Data in Brief/4 Visual Data- Splited/Document 4/4-1 rec14.jpg]

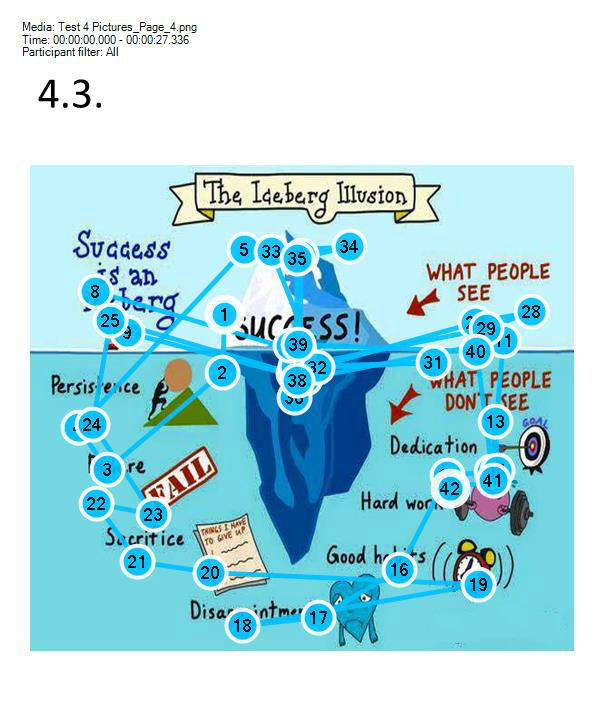

Supplement: Multimedia component 1 [file mmc1.zip › Data Data in Brief/4 Visual Data- Splited/Document 4/4-1 rec15.jpg]

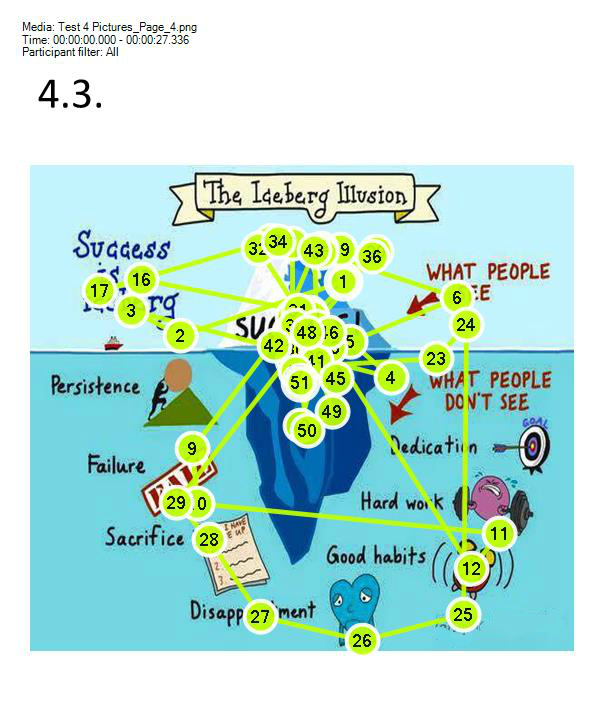

Supplement: Multimedia component 1 [file mmc1.zip › Data Data in Brief/4 Visual Data- Splited/Document 4/4-1 rec16.jpg]

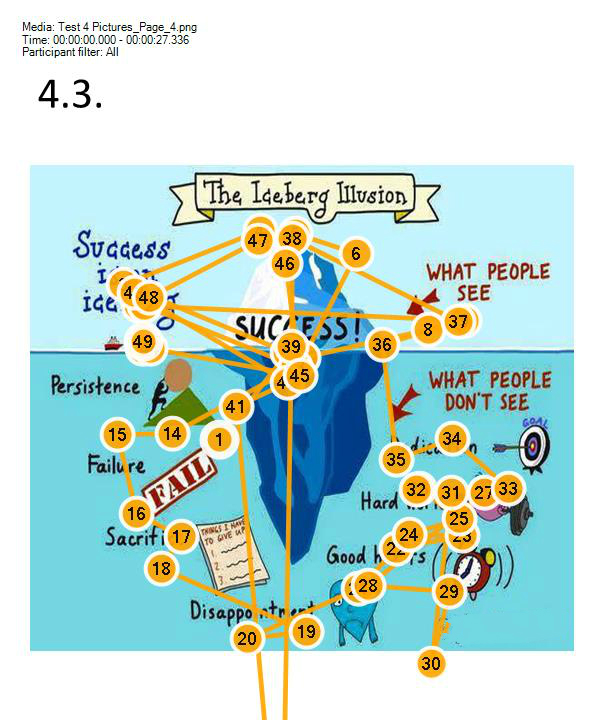

Supplement: Multimedia component 1 [file mmc1.zip › Data Data in Brief/4 Visual Data- Splited/Document 4/4-1 rec17.jpg]

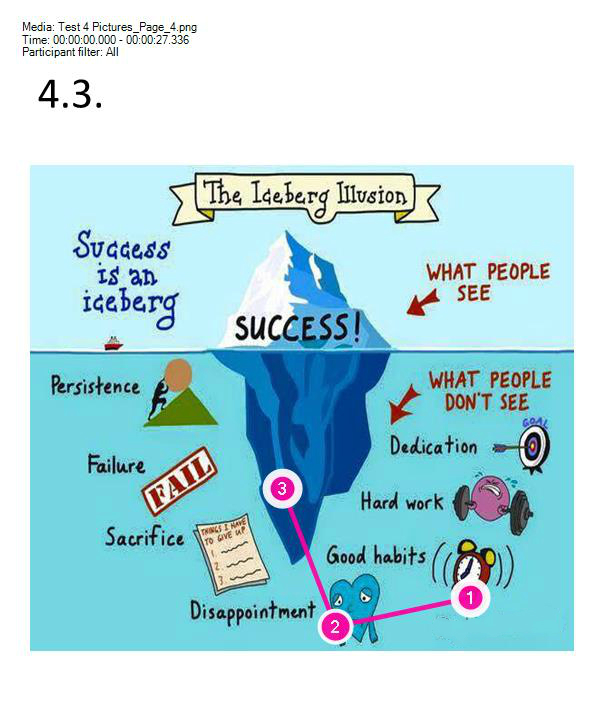

Supplement: Multimedia component 1 [file mmc1.zip › Data Data in Brief/4 Visual Data- Splited/Document 4/4-1 rec18.jpg]

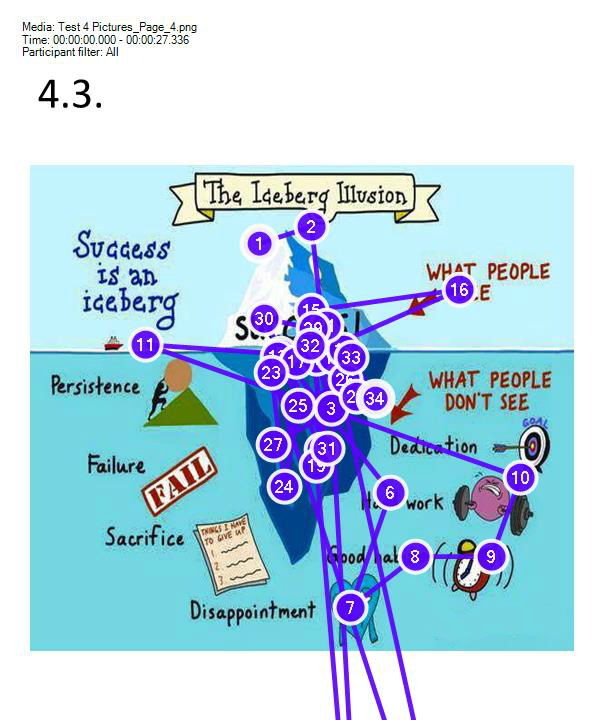

Supplement: Multimedia component 1 [file mmc1.zip › Data Data in Brief/4 Visual Data- Splited/Document 4/4-1 rec19.jpg]

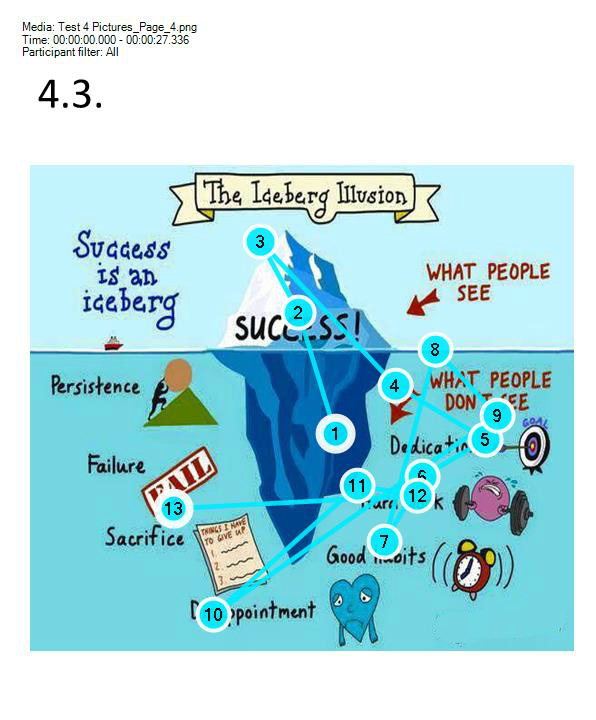

Supplement: Multimedia component 1 [file mmc1.zip › Data Data in Brief/4 Visual Data- Splited/Document 4/4-1 rec20.jpg]

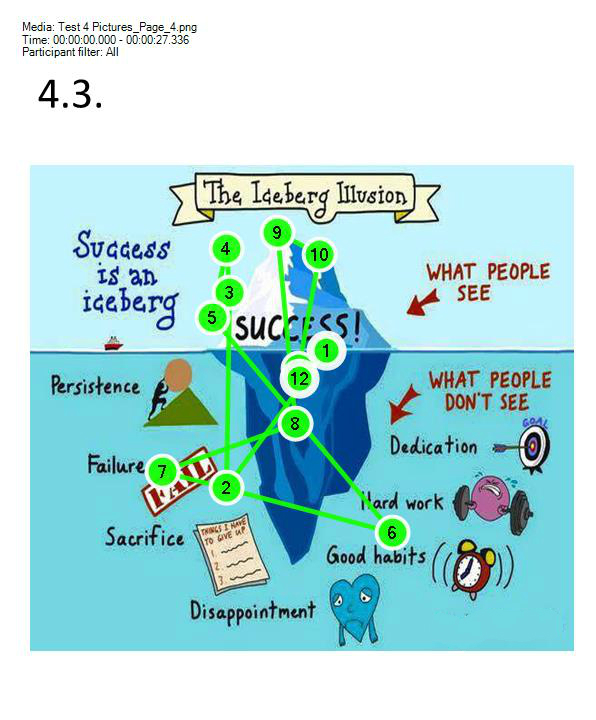

Supplement: Multimedia component 1 [file mmc1.zip › Data Data in Brief/4 Visual Data- Splited/Document 4/4-1 rec21.jpg]

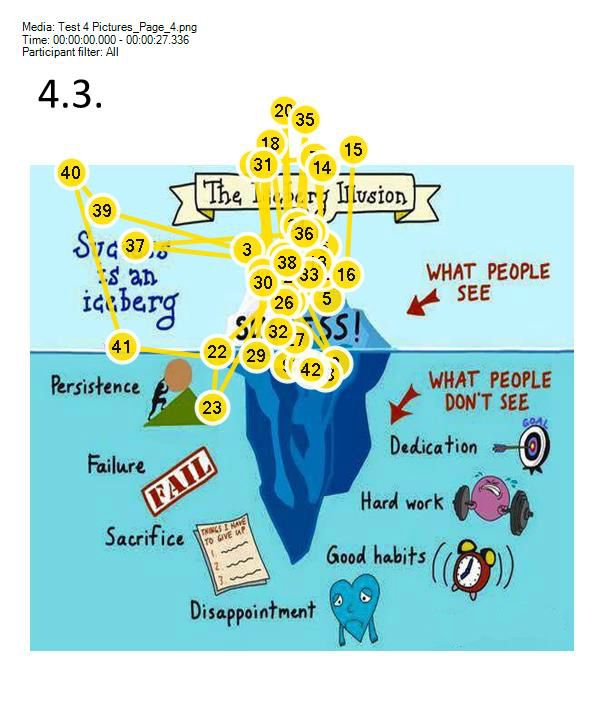

Supplement: Multimedia component 1 [file mmc1.zip › Data Data in Brief/4 Visual Data- Splited/Document 4/4-1 rec22.jpg]
